# Dopamine signaling governs macrophage-mediated acute lung injury through JAML/IL-10-coupled mitochondrial regulation

**DOI:** 10.1186/s12974-026-03823-1

**Published:** 2026-04-23

**Authors:** Di Wu, Ximing Liao, Jing Gao, Muyun Wang, Linlin Meng, Wujian Xu, Yanan He, Qian Zhang, Qiang Li, Kun Wang, Wei Gao

**Affiliations:** 1 Department of Pulmonary and Critical Care Medicine, Shanghai East Hospital, School of Medicine, Tongji University, Shanghai, 200092 China; 2 Second Department of Respiratory and Critical Care Medicine, The Fourth People’s Hospital of Jinan, Jinan, 250031 China; 3 Department of Respiratory and Critical Care Medicine, the Second People’s Hospital of Changzhou, the Third Affiliated Hospital of Nanjing Medical University, Changzhou, 213164 China; 4 Changzhou Medical Center, Nanjing Medical University, Changzhou, 213164 China

**Keywords:** Dopaminergic signaling, Acute lung injury, Macrophage, Junctional adhesion molecule-like protein, Mitochondrial protection

## Abstract

Acute lung injury (ALI) and acute respiratory distress syndrome (ARDS) remain devastating clinical entities characterized by uncontrolled pulmonary inflammation driven by dysregulated macrophage activation, with limited therapeutic options and high mortality. Emerging evidence implicates neuroimmune crosstalk as a pivotal regulator in inflammatory disorders, yet the role of dopaminergic signaling in orchestrating macrophage function during ALI remains ill-defined. Herein, we systematically characterized the dynamic perturbations of pulmonary dopaminergic signaling during ALI/ARDS progression and delineated the anti-inflammatory and cytoprotective properties of dopamine (DA) D1-like receptor (D1R) signaling in ALI mouse model and targeted macrophages. Mechanistically, DA-D1R activation mitigated macrophage hyperactivation by reversing lipopolysaccharide-induced mitochondrial dysfunction, thereby curbing excessive M1 polarization and maintaining cellular homeostasis. Transcriptomic profiling identified junctional adhesion molecule-like protein (JAML) as a critical downstream effector of the D1R agonist SKF38393 (SKF) in macrophages. SKF downregulated JAML expression and its interaction with interleukin (IL)-10, thus enhancing IL-10 bioavailability to sustain mitochondrial integrity and limit oxidative damage. Notably, the anti-inflammatory capacity of DA bioactivity system was validated in macrophages from ARDS patients and healthy controls, underscoring its translational potential. Collectively, our findings unravel a previously unrecognized DA-D1R-JAML/IL-10-mitochondria axis that governs macrophage-mediated ALI, positioning dopaminergic signaling as a promising therapeutic target for ARDS and other inflammatory disorders involving neuroimmune dysfunction.

**Graphical Abstract:**

**Figure:**
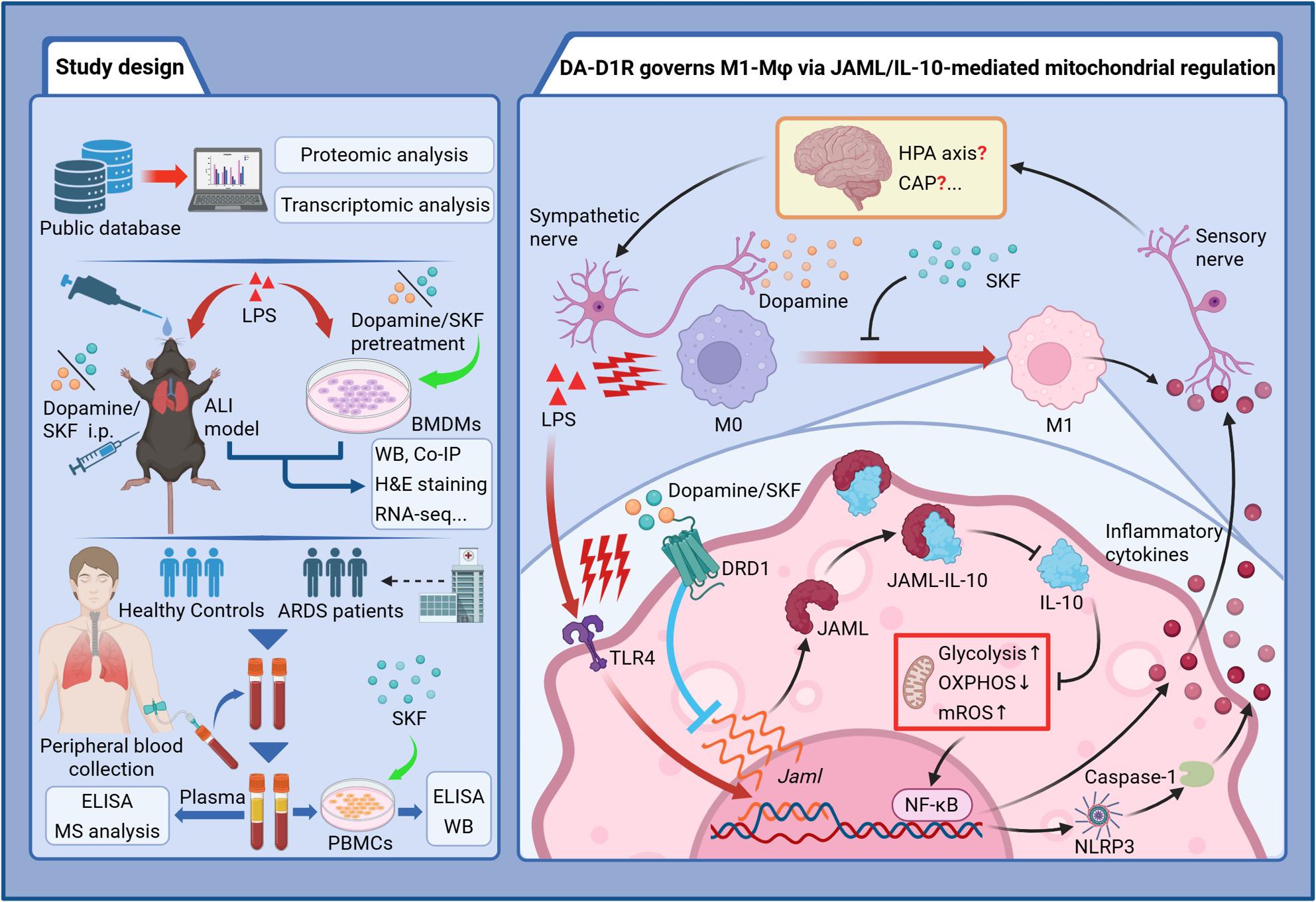
ToC text: The study design and the mechanism by which DA signaling acting on D1R regulates M1 macrophages (M1-Mφ) via JAML/IL-10-mediated mitochondrial regulation in ALI/ARDS.

**Supplementary Information:**

The online version contains supplementary material available at 10.1186/s12974-026-03823-1.

## Introduction

Acute lung injury (ALI) and its severe manifestation, acute respiratory distress syndrome (ARDS), are common clinical syndrome of acute hypoxemic respiratory failure due to diffuse alveolar injury and lung oedema [1]. The limited pharmacotherapeutic approaches for ALI is attributed to its intricate pathophysiology, involving the dysregulation of multiple overlapping and interconnected pathways governing inflammatory, coagulative, and tissue damage. Among the driving factors, macrophages, one of the key immune cells within the lung, play an essential role in neutrophilic inflammation and alveolar-capillary barrier disruption in ALI pathogenesis [2, 3]. Pro-inflammatory macrophage polarization (M1) coincides with profound mitochondrial dysfunction characterized by impaired oxidative phosphorylation (OXPHOS), fragmentation of mitochondrial networks, and reactive oxygen species (ROS) overproduction [4]. This phenotype and functional rewiring fuels NLRP3 inflammasome hyperactivation, therefore amplifying pro-inflammatory cytokine storms (e.g., chemokine (C-C motif) ligand (CCL)2, interleukin (IL)-6, tumor necrosis factor (TNF)-α)-mediated alveolar damage [5]. Thus, targeting the mitochondrial-NLRP3 axis in macrophages represents a critical therapeutic strategy for ALI.

Due to the lung’s dense innervation, emerging evidence have implicated neuroimmune crosstalk as a master regulator of macrophage reprogramming, representing a pivotal therapeutic target in ALI [6]. Among sympathetic catecholamines, dopamine (DA) presents a uniquely compelling and strategic target for modulating the macrophage mitochondrial-NLRP3 axis in ALI. The classical catecholamines norepinephrine and epinephrine exert their pulmonary immunomodulatory effects predominantly through the β_2_-adrenergic receptor (β₂AR), the functionally dominant subtype in lung immune cells [7, 8], a pathway often associated with generalized immunosuppression [9]. DA, however, utilizes a separate and more complex system, engaging a dedicated family of dopamine receptors (DRs) on macrophages [10–12]. This receptor family encompasses subtypes that couple to opposing G proteins (e.g., Gαs for D1-like (DRD1/DRD5), Gαi for D2-like (DRD2-4)) [13], thereby activating divergent intracellular signaling cascades that form the basis for nuanced, context-dependent immune modulation. Critically, dopaminergic signaling directly engages key inflammatory cascades. It can suppress systemic inflammation through specific receptor-mediated pathways, such as the DRD5-ARRB2-PP2A axis that blocks TRAF6-NF-κB signaling [14]. Moreover, it exhibits a direct mechanistic interplay with the NLRP3 inflammasome, a cornerstone of ALI pathology, as evidenced by its capacity to inhibit NLRP3 activation [15]. This dual capacity to intercept both the NF-κB and NLRP3 pro-inflammatory hubs underscores a precise and potent regulatory role at the core of inflammatory cascades. Furthermore, this context-dependent duality exemplifies DA’s broader functional plasticity in reshaping macrophage polarization [16–21], offering the potential for immunomodulation tailored to the dynamic phases of ALI. Collectively, these features establish dopaminergic immunomodulation as a precise and potent regulatory mechanism, positioning it as a prime therapeutic strategy whose operational logic in ALI awaits elucidation.

While DA is known to exert anti-inflammatory effects, how dopaminergic receptor signaling reprograms macrophage metabolism to resolve ALI remains a fundamental gap. Our prior work demonstrated that the D1 receptor (D1R)-selective agonist SKF38393 (SKF) protects against lung injury by attenuating macrophage hyperinflammation [22], pinpointing D1R as a critical protective node. However, the metabolic reprogramming mechanism triggered by SKF/D1R activation remains uncharacterized. A central unanswered question is how this signaling restores mitochondrial homeostasis in macrophages.

In this study, we first characterized the dynamic perturbations of the dopaminergic signaling system during the progression of ALI/ARDS. Then, we systematically delineated the anti-inflammatory and protective roles of DA-D1R signaling in both ALI mouse model and the targeted macrophages, laying a robust foundation for subsequent mechanistic investigations. Mechanistically, DA signaling mitigated macrophage hyperinflammation by reversing lipopolysaccharide (LPS)-induced mitochondrial dysfunction, thereby restraining excessive M1 polarization and preserving cellular homeostasis. RNA sequencing (RNA-seq) identified junctional adhesion molecule-like protein (JAML) as a critical downstream target of the D1R agonist SKF in macrophages, while further validation confirmed that SKF downregulated JAML expression, which in turn diminished its interaction with IL-10. This reduction in JAML-IL-10 binding enhanced IL-10 bioavailability, enabling its actions in mitochondrial protection and inflammation resolution. Finally, we validated the anti-inflammatory capacity of the DA signaling in peripheral blood mononuclear cells (PBMCs) from both ARDS patients and healthy controls, highlighting its translational potential for ALI therapeutic intervention.

## Results

### Dynamic alterations of the dopaminergic signaling during acute lung inflammation

**Fig. 1:**
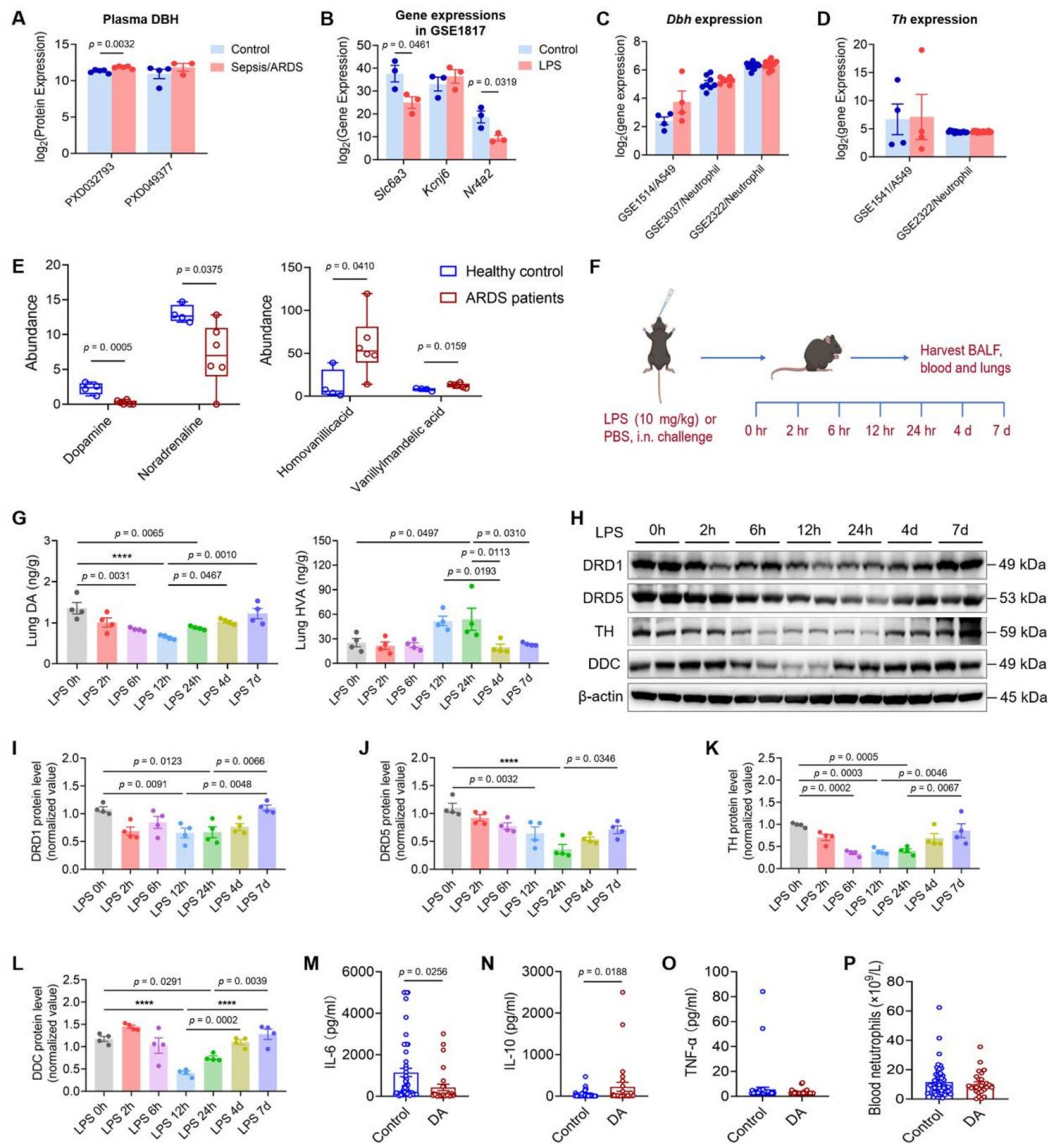
Reciprocal interactions between the dopaminergic signaling and pulmonary inflammation. **A** DBH level in plasma from healthy control and ARDS patients in public dataset PXD032793 and PXD049377 (*n* = 5, 5 in PXD032793; *n* = 4, 3 in PXD049377). **B-D** DA-related gene expression in different cells with or without LPS stimulation in GSE1817 (mouse lung tissue), GSE1514 (human A549 lung epithelial cell line), GSE3037 (peripheral blood neutrophils from septic patients), and GSE2322 (peripheral blood neutrophils from healthy volunteers) (**B**, *n* = 3 in each group of GSE1817; **C**, *n* = 4, 4 in GSE1514, *n* = 8, 8 in GSE3037, *n* = 13, 18 in GSE2322; **D**, *n* = 4, 4 in GSE1514, *n* = 13, 18 in GSE2322). **E** UPLC-ESI-MS/MS analysis was applied for neurotransmitters (DA, norepinephrine) and their metabolites (HVA, VMA) detection in plasma collected from healthy control and ARDS patients without any exogenous DA treatment (*n* = 4, 6). **F** Experimental strategy for dynamic analysis of dopaminergic signaling after LPS exposure (10 mg/kg) at 0, 2, 6, 12, 24 h and 4, 7 days. **G** Levels of DA and its major end metabolite HVA in lung tissue were analyzed using UPLC-ESI-MS/MS at different time points after LPS infusion (*n* = 4 in each group). **H-L** DRD1, DRD5 and the core key enzymes for DA synthesis TH and DDC were detected and analyzed (*n* = 4 in each group). **M-P** Productions of cytokines IL-6 (**M**), IL-10 (**N**), TNF-α (**O**), and neutrophil counts (**P**) in peripheral blood of ARDS patients without (*n* = 53 in control group) or with DA treatment (*n* = 28 in DA group). All samples were biologically independent and three or more independent experiments with similar results were performed (“n” represents the number of independent biological replicates). Data are presented as mean ± SEM and analyzed with a 95% confidence interval. Statistical analysis was performed using Mann-Whitney U-test (M, N), two-tailed unpaired Student t test or one-way ANOVA followed by Bonferroni’s post hoc test. ****p < 0.0001

To assess dopaminergic signaling activation during acute inflammation, we first analyzed public proteomic and transcriptomic databases using keywords “acute lung injury” and “acute respiratory distress syndrome”. After filtering datasets for dopaminergic protein/gene expression, we identified elevated DA β-hydroxylase (DBH) protein in peripheral blood plasma from ARDS patients compared to controls in ProteomeXchange Datasets (significant in PXD032793 and trend in PXD049377, Fig. 1A). For transcriptomics from Gene Expression Omnibus (GEO), analysis of the GSE1817 dataset (Fig. 1B) during LPS-induced acute inflammation revealed downregulation of the DA transporter gene *Slc6a3* and its regulator *Nr4a2*, alongside an upregulation trend in the synaptic release gene *Kcnj6*. Separately, analysis of datasets GSE1514/GSE3037/GSE2322 showed upward trends in tyrosine hydroxylase (*Th*), which governs dopamine biosynthesis, and *Dbh*, which converts dopamine to norepinephrine (Fig. 1C, D). In view of the above, we collected peripheral blood samples from ARDS patients who had not received any exogenous DA treatment and from matched healthy controls in our department (Table S1). Plasma metabolomics revealed significantly reduced DA and norepinephrine, and elevated homovanillic acid (HVA, the major end metabolite of DA) and vanillylmandelic acid (VMA, the major end metabolite of norepinephrine) in patients versus controls (Fig. 1E). Given that both *Th* and DBH expression show an increasing trend, it indicates an acceleration in DA synthesis and its conversion to norepinephrine. Meanwhile, the elevated levels of HVA and VMA provide direct evidence for a significant increase in the catabolism of the catecholamine pathway. Based on this, it is reasonable to infer that the low steady-state circulating levels of both DA and norepinephrine in ARDS patients are precisely due to their rapid synthesis and consumption at an elevated rate.

To further observe the dynamic changes of the dopaminergic signaling, we established a classical ALI mouse model and collected lung tissues at 0, 2, 6, and 12 h, and 4 and 7 days following LPS challenge for subsequent analyses (Fig. 1F). Consistent with clinical observations, DA levels in lung tissue decreased acutely within 6–24 h after LPS inhalation but recovered by day 4. Meanwhile, its end product HVA level increased sharply within 12–24 h and gradually returned to baseline after 4 days (Fig. 1G). Concordantly, the expression of pulmonary DRD1 and DRD5, as well as the core key enzymes in the DA synthesis pathway, TH and DOPA decarboxylase (DDC), decreased during the acute phase (12–24 h) and recovered by days 4–7 (Fig. 1H-L). In contrast, the expression of D2Rs (DRD2-4) showed no significant change after LPS challenge (Supplementary Fig. 1). Additionally, retrospective clinical data from our department confirmed functional relevance (Table S2): DA hydrochloride-treated ARDS patients had significantly lower IL-6 and higher IL-10 (Fig. 1M, N), and a downward trend in TNF-α, neutrophil counts (Fig. 1O, P) in peripheral blood compared to those without DA administration. The data collectively suggested dopaminergic pathway activation with anti-inflammatory effects in the early phase of ALI/ARDS.

### Anti-inflammatory effects of dopaminergic signaling in the ALI mouse model

**Fig. 2:**
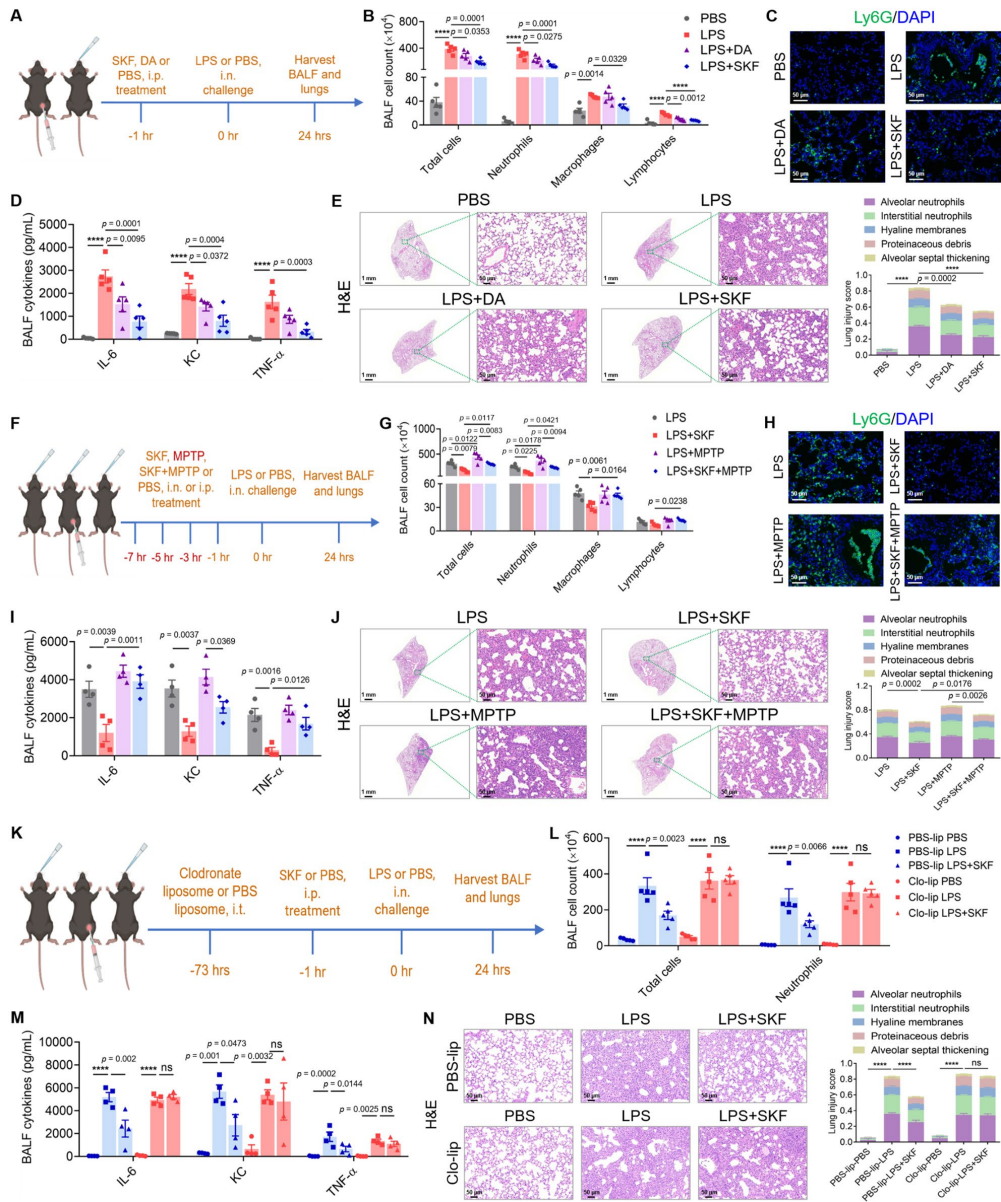
The anti-inflammatory effect of dopaminergic signaling on macrophage-driven ALI mouse model. **A** Schematic of the LPS-induced ALI mouse model, in which DA (300 µg per mouse), SKF (5 mg/kg), or an equal volume of DMSO in PBS (solvent control) was intraperitoneally injected 1 h prior to intranasal instillation of LPS (10 mg/kg) or sterile PBS (sham control). **B** Total cell number, neutrophil, macrophage and lymphocyte count in BALF of mice from distinct groups (*n* = 5 in each group). **C** Immunofluorescence image showing neutrophil accumulation and activation in the lung of ALI mice. Scale bar: 50 μm. **D** Production of cytokines in BALF was evaluated for IL-6, KC and TNF-α (*n* = 5 in each group). **E** Representative images of H&E-stained lung tissues and their injury score analysis at 24 h after LPS (*n* = 5 in each group). Scale bar: 1 mm in the left panel, 50 μm in the right panel. **F** Illustration of dopaminergic neuron ablation by MPTP in the ALI model. Specifically, MPTP or an equal volume of DMSO in PBS was delivered intranasally 4 times at 2-h intervals (15 mg/kg per dose); the last dose was co-administered with SKF (5 mg/kg intraperitoneally) or an equal volume of DMSO in PBS, and LPS (10 mg/kg) or sterile control PBS was intranasally instilled 1 h later. **G** Total cell number, neutrophil, macrophage and lymphocyte count in BALF of mice from distinct groups (*n* = 5 in each group). **H** Immunofluorescence image showing neutrophil accumulation and activation in the lung of ALI mice. Scale bar: 50 μm. **I** Production of cytokines in BALF was evaluated for IL-6, KC and TNF-α (*n* = 4 in each group). **J** Representative images of H&E-stained lung tissues and their injury score analysis at 24 h after LPS (*n* = 5 in each group). Scale bar: 1 mm in the left panel, 50 μm in the right panel. **K** Schematic diagram of Clo-lip-mediated depletion of pulmonary macrophages in the ALI mouse model. Clo-lip or PBS-lip (5 mg/mL, 75 µL per mouse intratracheally) were given 73 h before LPS, and SKF (5 mg/kg), or an equal volume of DMSO in PBS was intraperitoneally injected 1 h prior to intranasal instillation of LPS (10 mg/kg) or sterile PBS. **L** Total cell number and neutrophil count in BALF of mice from distinct groups (*n* = 5 in each group). **M** Production of cytokines in BALF was evaluated for IL-6, KC and TNF-α (*n* = 4 in each group). **N** Representative images of H&E-stained lung tissues and their injury score analysis at 24 h after LPS (*n* = 5 in each group). Scale bar: 50 μm. All samples were biologically independent and three or more independent experiments with similar results were performed (“n” represents the number of independent biological replicates). Data are presented as mean ± SEM and analyzed with a 95% confidence interval. Statistical analysis was performed via one-way ANOVA followed by Bonferroni’s post hoc test. ****p < 0.0001, ns, not significant

To evaluate dopaminergic protection against lung inflammation, we established a classic ALI mouse model (Fig. 2A). Intranasal LPS administration triggered robust neutrophilic inflammation, evidenced by massive inflammatory cell (especially neutrophil) infiltration in bronchoalveolar lavage fluid (BALF) and excessive Ly6G⁺ neutrophil accumulation in lung tissue (Fig. 2B, C; Supplementary Fig. 2A). It also induced elevated pro-inflammatory mediator (IL-6, KC and TNF-ɑ) production and lung histopathological damage (Fig. 2D, E). Both DA and D1R agonist SKF conferred significant protection in the ALI mice by reducing airway inflammatory cell, especially neutrophil infiltration, suppressing BALF cytokine secretion, and attenuating lung tissue damage, with SKF exhibiting a more pronounced efficacy (Fig. 2B-E). Consistently, SKF was demonstrated to restrain the activation of pro-inflammatory NLRP3/Caspase-1 pathway in ALI lungs, while DA elicited a comparatively weaker inhibitory effect on NLRP3 expression (Supplementary Fig. 2B, C).

To further verify whether dopaminergic signaling leads to the inhibition of LPS-induced inflammatory response in the lung, mice were intranasal administrated with neurotoxin 1-methyl-4-phenyl-1,2,3,6-tetrahydropyridine (MPTP) to ablate dopaminergic neurons [23], which showed decreased DA abundance within the lungs (Fig. 2F; Supplementary Fig. 3A). As seen in Fig. 2G-J and Supplementary Fig. 3B-D, SKF, as a direct D1R agonist, exerted its anti-inflammatory effects through a mechanism independent of intact pulmonary dopaminergic neurons. However, the observed attenuation of its protective efficacy in the LPS + SKF+MPTP group (compared to LPS + SKF) might be attributed to a profoundly altered inflammatory milieu. Specifically, MPTP-induced acute neuronal death could release damage-associated molecular patterns, which trigger a robust, non-specific “inflammatory storm”. This was evidenced by the exacerbated inflammation in the LPS+MPTP group relative to LPS alone. Within this background, the relatively fine-tuned anti-inflammatory signal mediated by SKF through D1R is not abolished but is partially overwhelmed or masked.

Based on our previous study [22], we consider that macrophages are one of the key target cells for DA signal to exert its anti-inflammatory effect in ALI. Accordingly, we depleted pulmonary macrophages via intratracheal clodronate liposome (Clo-lip) instillation [24]. Mice received Clo-lip 72 h prior to SKF administration, followed by LPS challenge 1 h post-SKF (Fig. 2K). Blank liposome (PBS-lip) served as vehicle control at the same volume. After 24 h of LPS stimulation, the BALF and lungs were collected to assess the depletion efficiency of macrophages. As shown in Supplementary Fig. 4A, B, Clo-lip administration led to dramatic reduction in lung macrophages in both steady state and inflammatory condition, verifying the effective removal of pulmonary macrophages. In the case, macrophage-depleted mice showed abolition of SKF’s suppression of LPS-induced airway neutrophilia and cytokine overproduction, as well as histopathological damage and pro-inflammatory signaling activation in lung tissues (Fig. 2L-N; Supplementary Fig. 4C-E). Altogether, these data establish lung macrophage as essential mediator of the anti-inflammatory efficacy of DA signaling in ALI. In addition, dopaminergic neurons were presumably implicated in this regulatory process.

### D1R mediates the protective effects of DA against macrophage overactivation and lung neutrophilic inflammation

**Fig. 3:**
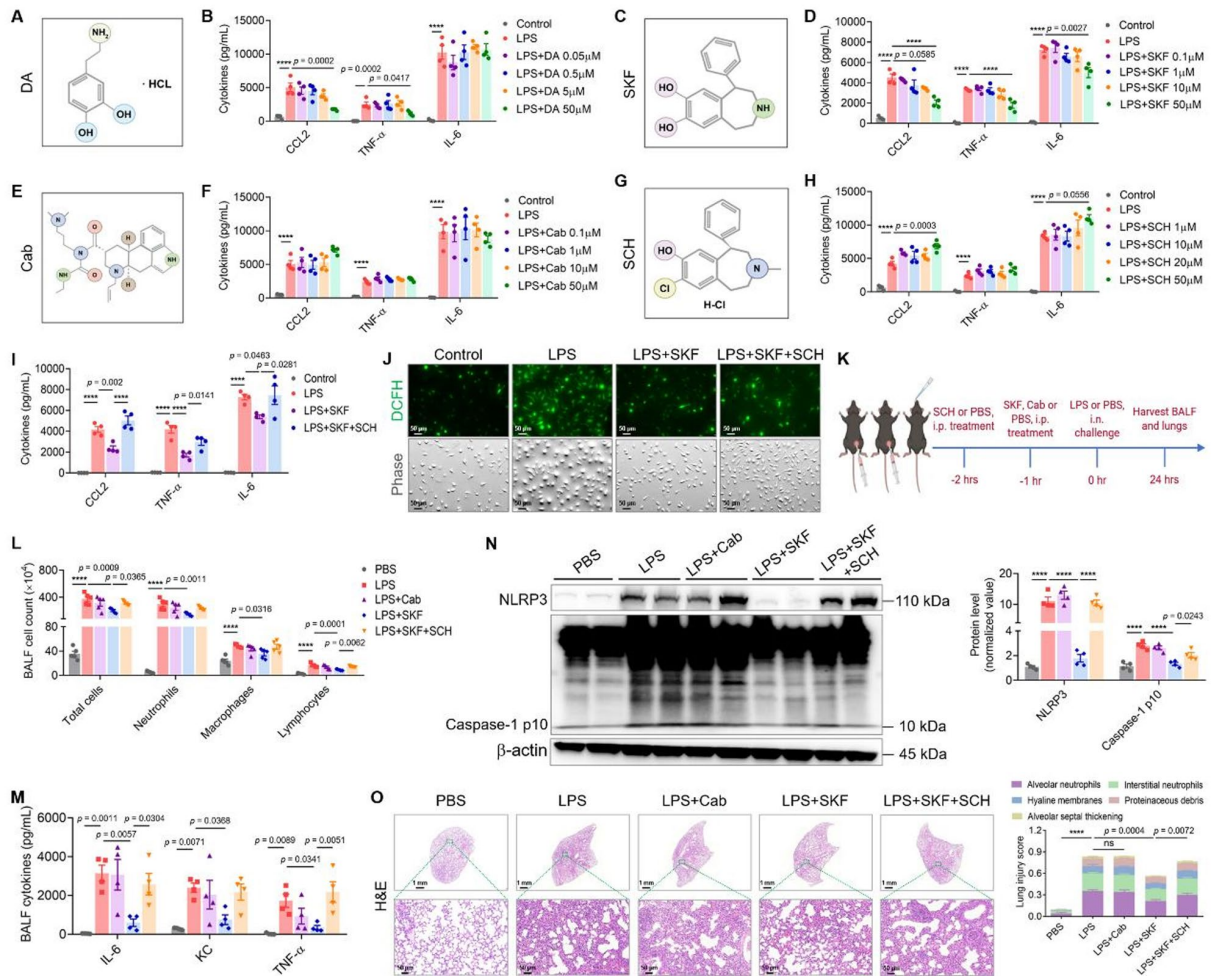
Dopaminergic signaling suppresses lung inflammation via D1R signaling. **A-I** Schematic diagrams of the chemical structures of DA (**A**), SKF (**C**), Cab (**E**), and SCH (**G**); BMDMs were treated with DA (0.05 µM, 0.5 µM, 5 µM, 50 µM), SKF (0.1 µM, 1 µM, 10 µM, 50 µM), Cab (0.1 µM, 1 µM, 10 µM, 50 µM), SCH (1 µM, 10 µM, 20 µM, 50 µM), or an equal volume of DMSO in medium 1 h prior to stimulation with LPS (100 ng/mL) or equal volume of medium. After 24 h of culture, the supernatants were collected for the detection of CCL2, TNF-α, IL-6 secretion (**B**,** D**,** F**,** H**,** I**) (*n* = 4 in each group). **J** Representative pictures of intracellular ROS staining with DCFH-DA probe, shown in green, and their corresponding brightfield images. Scale bar: 50 μm. *n* = 4 in each group. In panels **I** and **J**, the concentrations of SKF and SCH were 50 µM and 10 µM, respectively. **K** Schematic of the LPS-induced ALI mouse model, in which SCH (0.5 mg/kg) or an equal volume of DMSO in PBS was intraperitoneally injected 2 h, while SKF (5 mg/kg), Cab (5 mg/kg) or an equal volume of DMSO in PBS was administrated intraperitoneally 1 h prior to intranasal instillation of LPS (10 mg/kg) or sterile PBS. **L** Total cell number, neutrophil, macrophage and lymphocyte count in BALF of mice from distinct groups (*n* = 5 in each group). **M** Production of cytokines in BALF was estimated for IL-6, KC and TNF-α (*n* = 4 in each group). **N** The activation of pro-inflammatory NLRP3/Caspase-1 pathway in lung tissues of different mice. **O** Representative images of H&E-stained lung tissues and their injury score analysis at 24 h after LPS (*n* = 5 in each group). Scale bar: 1 mm in the upper panel, 50 μm in the below panel. All samples were biologically independent and three or more independent experiments with similar results were performed (“n” represents the number of independent biological replicates). Data are presented as mean ± SEM and analyzed with a 95% confidence interval. Statistical analysis was performed via one-way ANOVA followed by Bonferroni’s post hoc test. ****p < 0.0001, ns, not significant

DA receptors belong to metabotropic G protein-coupled receptors and consist of five major subtypes, designated as D1R (DRD1, DRD5) and D2R (DRD2-4). Their expression and function in immune cells vary by cellular context and tissue microenvironment. To identify receptor-specific anti-inflammatory mechanism, we collected mouse bone marrow-derived macrophages (BMDMs) and treated them with DA and SKF, a potent agonist of D1R. Under LPS stimulation, DA (50 µM) significantly suppressed the release of pro-inflammatory cytokines CCL2 and TNF-α but not IL-6, while SKF at the same concentration could inhibit the secretion of all cytokines (Fig. 3A-D). In contrast, D2R agonist Cabergoline (Cab) failed to show any significant effects in LPS-stimulated BMDMs (Fig. 3E, F).

To further determine the function of D1R in DA-mediated macrophage suppression, we applied a potent antagonist of D1R, SCH23390 (SCH) [25]. The results demonstrated that SCH could largely abrogate the inhibitory effects of both SKF and DA on macrophage overactivation (Fig. 3G-I; Supplementary Fig. 5G, H). We stated that the pro-inflammatory effect of a high dose of SCH (50 µM) observed in Fig. 3H might result from its blockade of a constitutive autocrine dopaminergic tone in macrophages. This hypothesis is grounded in established evidence that macrophages can synthesize dopamine, which acts on their D1R to maintain a baseline anti-inflammatory state [26]. By antagonizing these receptors, SCH could disrupt this tonic inhibition. Notably, such effects were unrelated to the cytotoxicity of these drugs (Supplementary Figure. 5 A-F). The uncontrolled inflammatory response upon LPS challenge was attributed to excessive intracellular ROS. Accordingly, we evaluated the effects of D1R activation on oxidative damage. Using the 2’,7’-dichlorodihydrofluorescein diacetate (DCFH-DA) fluorescent probe, our results revealed that SKF significantly decreased LPS-induced ROS production with dimmer green fluorescence and lower fluorescence intensity, which could be reversed by SCH pre-treatment (Fig. 3J; Supplementary Fig. 5I).

We next analyzed lung inflammation in ALI mice by use of SKF, Cab and SCH to further elucidate the role of D1R activation (Fig. 3K). Mice administrated with SKF, but not Cab, exhibited reduced airway inflammation and lung tissue damage upon LPS stimulation (Fig. 3L-O; Supplementary Fig. 6). SCH pre-treatment significantly attenuated SKF-mediated suppression of local inflammatory cell infiltration in lungs of ALI mice (Fig. 3L; Supplementary Fig. 6). Concurrently, it abrogated SKF-induced decrease in the production of IL-6, TNF-ɑ in BALF and the activation of NLRP3/Caspase-1 pathway in lung tissues (Fig. 3M, N). As the consequence of augmented inflammatory response, exacerbated tissue damage in ALI mice administered with SCH was confirmed by histological staining (Fig. 3O). Taken together, these findings indicated that D1R-mediated DA signaling suppressed macrophage overactivation in vitro, which likely contributed to the overall restriction of neutrophilic inflammation observed in the lung during ALI.

### D1R activation by SKF constrains M1 macrophage polarization through mitochondrial reprogramming

**Fig. 4:**
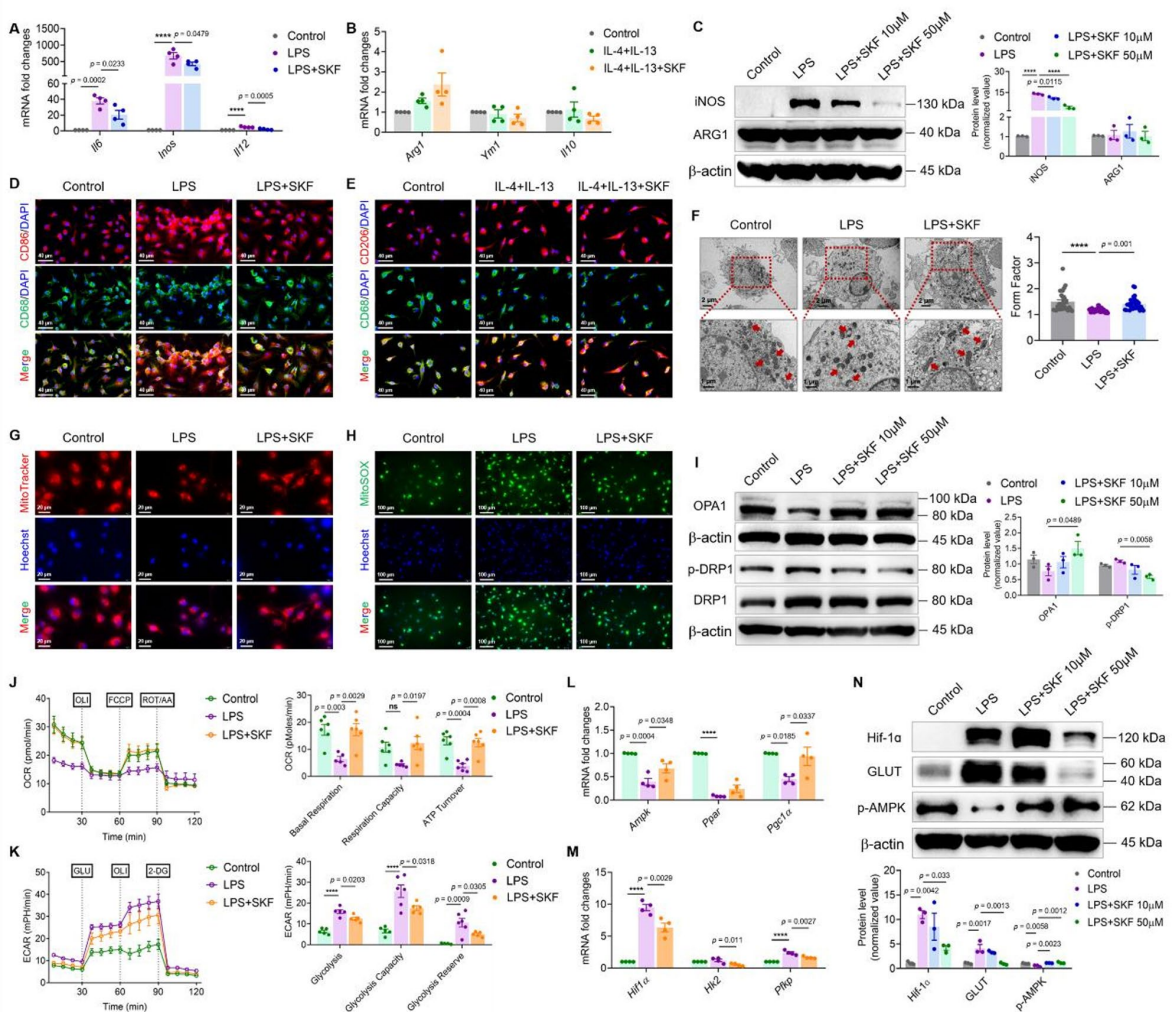
SKF inhibits M1 polarization and maintains mitochondrial homeostasis. **A**,** B** Expressions of M1 markers *Il6*, *Inos*, *Il12 *(**A**), and M2 markers *Arg1*, *Ym1*, *Il10 *(**B**) in BMDMs subjected to 50 µM SKF or an equal volume of DMSO in medium 1 h prior to stimulation with LPS (100 ng/mL), IL-4 + IL-13 (20 ng/mL) or equal volume of medium were quantified by qPCR (*n* = 4 in each group). **C** Expressions of M1 marker iNOS and M2 marker ARG1 in BMDMs subjected to different treatments were identified via immunoblotting assay (*n* = 3 in each group). **D**,** E** Immunofluorescence staining with M1 marker CD68/CD86 (**D**) and M2 marker CD68/CD206 (**E**) in BMDMs under distinct stimulations as described in (**A**) and (**B**). Nuclei were stained with DAPI (4′, 6-Diamidino-2-phenylindole), displayed in blue. Scale bar: 40 μm. **F** Representative TEM images and form factor analysis of the mitochondria morphological parameter in BMDMs under different treatments. Form Factor = Perimeter² / (4π × Area). Red arrowheads indicated the mitochondria. Scale bar: 2 μm in the upper panel, 1 μm in the below panel. *n* = 30 in each group. **G**,** H** Representative images of MitoTracker (**G**) and MitoSOX (**H**) probe-stained BMDMs after distinct treatments. The nuclei were stained with Hoechst, displayed in blue. Scale bars, 20 μm in MitoTracker, 100 μm in MitoSOX. **I** Expression of fusion protein OPA1 and fission protein DRP1 in BMDMs subjected to different treatments were identified by immunoblotting assay (*n* = 3 in each group). **J**,** K** OCR (**J**, *n* = 6, 5, 6 for basal respiration; *n* = 6, 5, 6 for respiration capacity; *n* = 6, 6, 6 for ATP turnover) and ECAR (**K**, *n* = 5, 5, 5 for glycolysis; *n* = 5, 6, 5 for glycolysis capacity; *n* = 5, 6, 5 for glycolysis reserve) of mouse BMDMs with different interventions were measured with a Seahorse XFe96 analyser. **L**,** M** Expression of OXPHOS-related genes *Ampk*, *Ppar*, *Pgc1α *(**L**), and glycolysis-related genes *Hif1α*, *Hk2*, *Pfkp *(**M**) in BMDMs subjected to distinct treatments were quantified by qPCR (*n* = 4 in each group). **N** Expression of Hif-1α, GLUT and p-AMPK in BMDMs were determined using immunoblotting assay (*n* = 3 in each group). All samples were biologically independent and three or more independent experiments with similar results were performed (“n” represents the number of independent biological replicates). Data are presented as mean ± SEM and analyzed with a 95% confidence interval. Statistical analysis was performed via one-way ANOVA followed by Bonferroni’s post hoc test. ****p < 0.0001, ns, not significant

Existing evidence indicated that constraining macrophage commitment to pro-inflammatory M1 phenotype may be a promising therapeutic strategy for ALI/ARDS and other inflammatory diseases [27, 28]. As DA-D1R signaling primarily targeted lung macrophage to alleviate ALI (Fig. 2) and reshaped the cytokine production profile in LPS-stimulated BMDMs (Fig. 3), we speculated that D1R activation could suppress M1 macrophage polarization to reduce inflammatory response. To test this hypothesis, mouse BMDMs were collected and cultured to investigate the impact of SKF on macrophage polarization ex vivo. BMDMs can polarize into functionally distinct phenotypes in response to various stimuli, predominantly the pro-inflammatory M1 and anti-inflammatory M2 subtypes. As shown in Fig. 4A, LPS elevated the pro-inflammatory cytokines IL-6, iNOS and IL-12 expression, which were mainly produced by M1 macrophages and reduced by SKF treatment. However, during M2 polarization induced by IL-4 and IL-13 co-stimulation, SKF did not affect the gene expression of the M2 markers ARG1, YM1 and IL-10 (Fig. 4B). At protein expression level, the M1 marker iNOS, rather than the M2 marker ARG1, was significantly downregulated by SKF under LPS challenge (Fig. 4C). By analyzing the characteristic molecular of M1 (CD86) and M2 (CD206) macrophages using immunofluorescence, we found that SKF reduced the red fluorescence intensity of CD86 in BMDMs under LPS stimulation, whereas had no obvious effect on the fluorescence intensity of CD206 in cells co-stimulated with IL-4 and IL-13 (Fig. 4D, E; Supplementary Fig. 7A, B). Notably, IL-4/IL-13 co-stimulation failed to augment the expression of M2 gene markers simply because the inherent M2-biased polarization state of macrophage colony-stimulating factor-derived BMDMs prior to cytokine exposure [29, 30].

Given that macrophage polarization is tightly coupled to metabolic reprogramming [5], we next explored whether SKF modulates mitochondrial function to regulate M1 polarization. We first observed the mitochondrial morphology by transmission electron microscopy (TEM). At 24 h following LPS exposure, swollen mitochondria with cristae loss were seen in BMDMs, whereas such structural alterations were minimal in the SKF-treated group (Fig. 4F). Quantitative assessment of mitochondrial function via MitoTracker and MitoSOX staining confirmed SKF-mediated bioenergetic enhancement. As revealed in Fig. 4G and Supplementary Fig. 7C, LPS stimulation diminished MitoTracker fluorescence, indicating mitochondrial damage, which could be enhanced by SKF addition. Similarly, SKF reversed LPS-elevated MitoSOX fluorescence in BMDMs, representing the potential mitochondrial ROS (mROS) scavenging capacity of SKF (Fig. 4H; Supplementary Fig. 7D). Mitochondrial homeostasis, critical for energy metabolism, is tightly modulated by fission-fusion dynamics [31]. We next assessed expression of fusion protein OPA1 and fission protein DRP1, and found that SKF markedly alleviated LPS-induced OPA1 reduction and DRP1 phosphorylation in BMDMs, indicating the improved mitochondrial dynamics by D1R activation (Fig. 4I). As consequences of mitochondrial dysfunction, decreased OXPHOS and increased glycolysis were demonstrated in BMDMs under LPS challenge via seahorse assay. Specifically, LPS impaired the basal oxygen consumption rate (OCR) and ATP turnover, and promoted glycolytic function in macrophages, which were reversed by SKF (Fig. 4J, K). Consistent with these observations, SKF also increased the expression of key regulators (AMPK, PGC-1α) for OXPHOS pathway, while downregulated glycolysis-related factors Hif-1α, HK2, PFKP and GLUT in LPS-stimulated BMDMs at transcriptional or protein levels (Fig. 4L-N). Collectively, these findings suggested that D1R activation may constrain exaggerated pro-inflammatory M1 macrophage polarization via mitochondrial-metabolic optimization.

### SKF targets JAML to exert anti-inflammatory effects

**Fig. 5:**
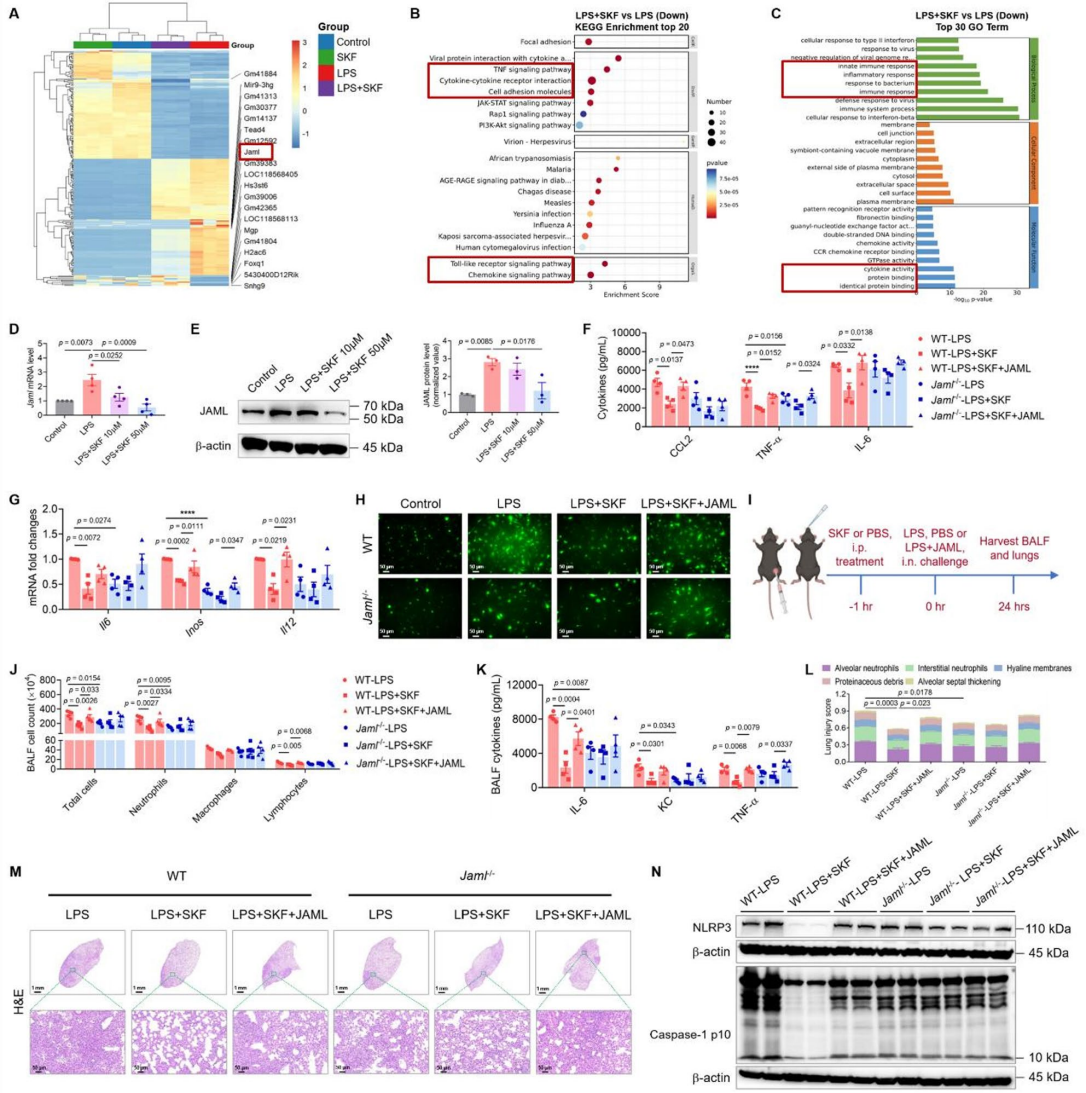
SKF exerts anti-inflammatory effect through targeting JAML. **A** A heatmap exhibiting the most different expressed genes (DEGs) between indicated four groups. **B**,** C** KEGG (**B**) and GO (**C**) enrichment analyses were performed on the DEGs between LPS + SKF versus LPS group, the most downregulated enriched terms were showed. *n* = 3 in each group. **D**,** E** The mRNA (**D**, *n* = 4 in each group) and protein (**E**, *n* = 3 in each group) expression of JAML was determined via qPCR and immunoblotting assay. **F** The effect of JAML expression on the secretion of pro-inflammatory cytokines CCL2, TNF-α, IL-6 by BMDMs stimulated with LPS (*n* = 4 in each group). Specifically, BMDMs from WT and *Jaml*^−/−^ mice were treated with SKF (50 µM), SKF combined with exogenous JAML protein (1 µg/mL), or an equal volume of DMSO-containing medium 1 h prior to LPS (100 ng/mL) stimulation. **G** Expressions of M1 markers *Il6*, *Inos*, *Il12* in BMDMs subjected to different treatments were quantified by qPCR (*n* = 4 in each group). **H** Representative pictures of intracellular ROS staining with DCFH-DA probe, shown in green. Scale bar: 50 μm. *n* = 4 in each group. **I** Schematic of JAML-modulated ALI mouse model. Specifically, SKF (5 mg/kg) or an equal volume of DMSO in PBS was intraperitoneally injected 1 h prior to intranasal instillation of LPS (10 mg/kg), exogenous JAML (100 µg/kg) concurrently with LPS, or sterile PBS. **J** Total cell number, neutrophil, macrophage and lymphocyte count in BALF of mice from distinct groups (*n* = 5 in each group). **K** Production of cytokines in BALF was estimated for IL-6, KC and TNF-α (*n* = 4 in each group). **L**,** M** Representative images of H&E-stained lung tissues (**M**) and their injury score analysis (**L**) at 24 h after LPS (*n* = 5 in each group). Scale bar: 1 mm in the upper panel, 50 μm in the below panel. **N** The activation of pro-inflammatory NLRP3/Caspase-1 pathway in lung tissues of different mice. All samples were biologically independent and three or more independent experiments with similar results were performed (“n” represents the number of independent biological replicates). Data are presented as mean ± SEM and analyzed with a 95% confidence interval. Statistical analysis was performed via one-way ANOVA followed by Bonferroni’s post hoc test. ****p < 0.0001

To profile the transcriptional status of macrophages that were modulated by SKF, RNA-seq was performed to analyze BMDMs treated with SKF, LPS alone, or together with SKF. A heatmap of all differentially expressed genes across the four groups was shown in Fig. 5A. Among these, several genes, including the gene for JAML, a critical immunoregulator in inflammatory diseases, were heavily upregulated under LPS treatment but suppressed by SKF. The Kyoto Encyclopedia of Genes and Genomes (KEGG) analysis identified overlapping key inflammatory pathways suppressed by SKF, including TNF signaling pathway, cytokine-cytokine receptor interaction, cell adhesion molecules, Toll-like receptor (TLR) signaling, and chemokine signaling (Fig. 5B). Besides, the gene ontology (GO) annotation revealed those downregulated genes by SKF were involved in innate immune response, inflammatory response, response to bacterium, immune response, cytokine activity, and protein binding (Fig. 5C). Based on the transcriptomic data, we further verified the inhibitory effect of SKF on the activation of the TLR4/NF-κB pathway in macrophages under LPS stimulation using immunoblotting analysis (Supplementary Fig. 8A). In addition, we examined the impact of SKF/DA on inflammasome activation and its downstream signaling events. Although SKF/DA did not affect the expression of pro-IL-1β, they significantly attenuated the cleavage of pro-IL-1β and pro-Caspase-1 into their mature forms (p17 and p10, respectively) in BMDMs stimulated with LPS plus ATP. Furthermore, they could also reduce the cleavage of gasdermin D into its active N-terminal fragment, which executes pyroptosis (Supplementary Fig. 8C, D). These results thus indicated DA-D1R exerts its global anti-inflammatory effect by concomitantly inhibiting two critical steps: TLR4/NF-κB-driven priming and the subsequent inflammasome-mediated pyroptotic cascade in macrophages.

Among several candidates identified by RNA-seq, JAML was prioritized for mechanistic study because its modulation most consistently and significantly altered the inflammatory phenotype, unlike other targets such as Tead4. JAML, a type I transmembrane glycoprotein found on the cell membrane, in the cytoplasm, and in a soluble form [32, 33], mediates leukocyte migration via binding intracellular partners [34, 35]. Its expression is particularly enriched and inducible in monocytes/macrophages and neutrophils [36, 37]. We demonstrated that LPS significantly elevated JAML expression in BMDMs, which could be abrogated by SKF at both mRNA and protein levels (Fig. 5D, E). To determine whether JAML served as a target for SKF, we employed wild type (WT) and *Jaml*^−/−^ mice with or without recombinant JAML treatment. The *Jaml* knockout demonstrated good efficiency and did not affect the baseline release of cellular pro‑inflammatory cytokines, thus laying a foundation for subsequent experiments (Supplementary Fig. 9A, B). As shown in Fig. 5F, JAML depletion not only attenuated LPS-induced TNF-α production, but also diminished the inhibitory effect of SKF on the cytokines. Moreover, exogenous JAML supplementation reversed the anti-inflammatory actions of SKF in BMDMs from WT mice. This pattern extended to M1 marker expression (Fig. 5G) and cellular ROS generation (Fig. 5H; Supplementary Fig. 9D), demonstrating the key role of JAML in SKF-mediated regulation in LPS-triggered inflammatory responses and oxidative stress. It is worth noting that neither endogenous JAML depletion nor its exogenous supplementation could significantly affect the viability of BMDMs during LPS exposure (Supplementary Fig. 9C).

Using an LPS-induced ALI mouse model established by intranasal instillation (Fig. 5I), we found that *Jaml* depletion not only alleviated lung inflammatory injury but also attenuated the protective effect of SKF, whereas supplementation with JAML reversed the suppressive effect of SKF on inflammatory responses in WT mice. These changes were evidenced by alterations in airway inflammatory cell infiltration, particularly neutrophils, pro-inflammatory cytokine secretion, tissue injury severity, and activation of the NLRP3/Caspase-1 pathway in lung tissue (Fig. 5J-N; Supplementary Fig. 9H, I). Of note, *Jaml* knockout itself did not affect the measured parameters in mice under physiological conditions (Supplementary Fig. 9E-G). Taken together, our computational and experimental analyses suggested that SKF profoundly affected the transcriptional status of macrophages under LPS stimulation, establishing JAML as a core mediator through which DA signaling restrains inflammatory cascades.

### JAML deletion amplifies IL-10 to drive SKF-dependent mitochondrial protection and inflammation resolution

**Fig. 6:**
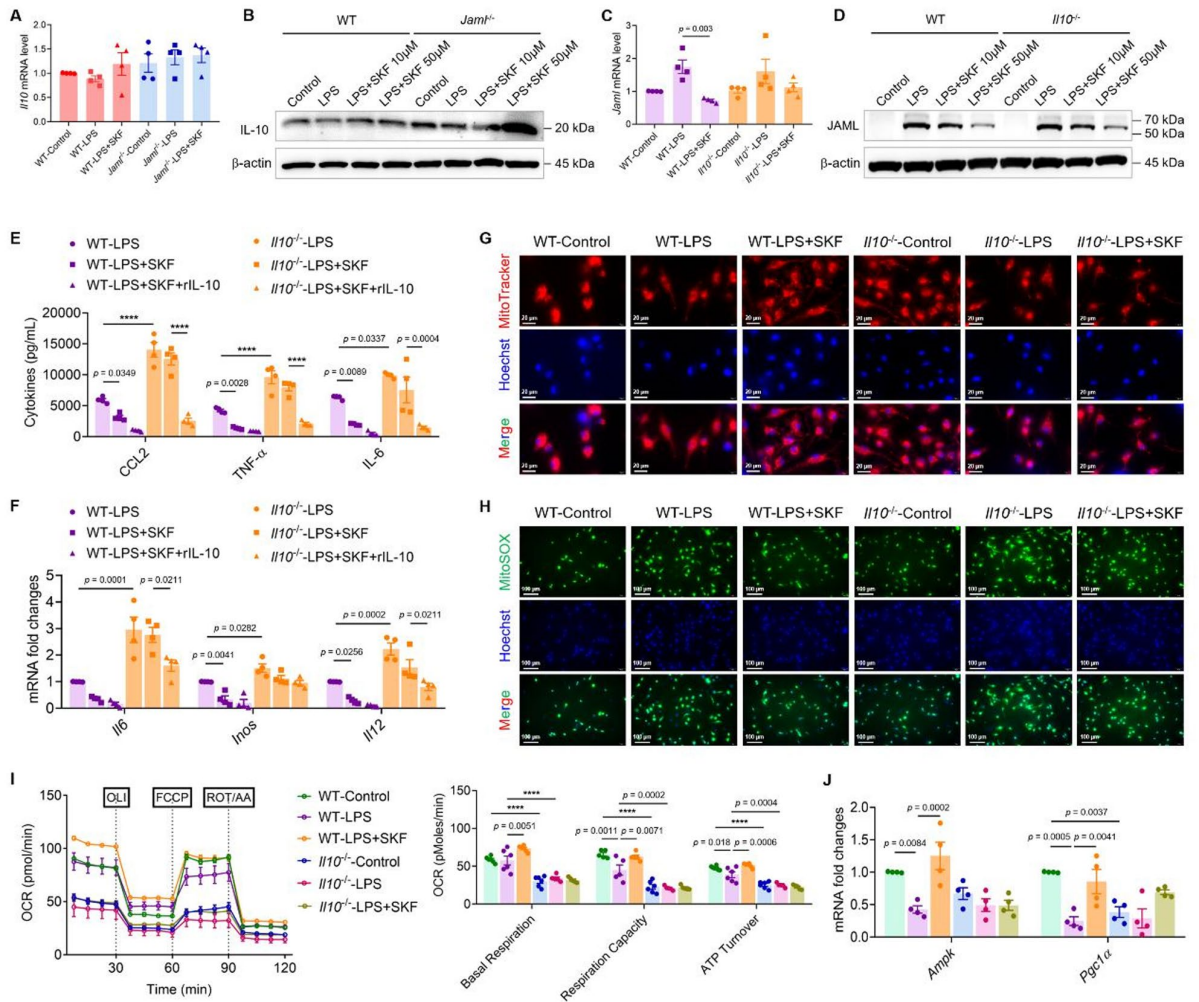
SKF-JAML regulates mitochondrial activity and inflammation via IL-10. **A**,** B** BMDMs from WT and *Jaml*^−/−^ mice were treated with SKF (10 µM, 50 µM), or an equal volume of DMSO-containing medium 1 h prior to LPS (100 ng/mL) stimulation. The mRNA (A, *n* = 4 in each group) and protein (**B**, *n* = 4 in each group**) **expression of IL-10 was determined by qPCR and immunoblotting assay. **C**,** D** BMDMs from WT and *Il10*^−/−^ mice were treated with SKF (10 µM, 50 µM), or an equal volume of DMSO-containing medium 1 h prior to LPS (100 ng/mL) stimulation. The mRNA (**C**, *n* = 4 in each group) and protein (**D**, *n* = 3 in each group) expression of JAML was estimated by qPCR and immunoblotting assay. **E**,** F **BMDMs from WT and *Il10*^−/−^ mice were treated with SKF (50 µM), SKF combined with rIL-10 (100 ng/mL), or an equal volume of DMSO-containing medium 1 h prior to LPS (100 ng/mL) stimulation. The effect of IL-10 on the secretion of pro-inflammatory cytokines CCL2, TNF-α, IL-6 (**E**, *n* = 4 in each group), and the expression of M1 markers *Il6*, *Inos*, *Il12* (**F**, *n* = 4, 4, 4, 4, 4, 5 for *Il6*, *Inos*; *n* = 4 in each group for *Il12*) by BMDMs stimulated with LPS. **G**,** H** Representative images of MitoTracker (**G**) and MitoSOX (**H**) probe-stained BMDMs under distinct treatments. The nuclei were stained with Hoechst, displayed in blue. Scale bars, 20 μm in MitoTracker, 100 μm in MitoSOX. **I** OCR of BMDMs from WT and *Il10*^−/−^ mice with different interventions was measured using a Seahorse XFe96 analyser. *n* = 6, 6, 6, 6, 5, 5 for basal respiration; *n* = 5, 5, 5, 6, 5, 5 for respiration capacity; *n* = 6, 6, 6, 6, 5, 5 for ATP turnover. **J** Expression of OXPHOS-related genes *Ampk*, *Pgc1* in BMDMs from WT and *Il10*^−/−^ mice subjected to distinct treatments was quantified by qPCR (*n* = 4 in each group). All samples were biologically independent and three or more independent experiments with similar results were performed (“n” represents the number of independent biological replicates). Data are presented as mean ± SEM and analyzed with a 95% confidence interval. Statistical analysis was performed via one-way ANOVA followed by Bonferroni’s post hoc test. ****p < 0.0001

Given that ARDS patients exhibited elevated serum IL-10 following DA therapy (Fig. 1N), which emerges as a master immunometabolic regulator [38], we investigated its contribution to the anti-inflammatory activity of SKF. In BMDMs derived from WT and *Jaml*^−/−^ mice, SKF (50 µM) elevated IL-10 protein, but not mRNA expression, an effect dramatically amplified by JAML ablation (Fig. 6A, B; Supplementary Fig. 10A, B). We next generated *Il10*^−/−^ mice to dissect the role of IL-10 in this modulatory axis (Supplementary Fig. 10C). However, IL-10 deficiency exerted no significant effect on either basal JAML expression or its responsiveness to SKF (Fig. 6C, D; Supplementary Fig. 10D, E). We thus speculated IL-10 as a downstream effector of the SKF-JAML axis and a master immunoregulator executing the anti-inflammatory actions of DA signaling. Compared to WT controls, IL-10 deficiency did not affect the baseline secretion of pro-inflammatory factors in BMDMs (Supplementary Fig. 10F). However, upon LPS stimulation, IL-10 knockout not only amplified the expression of pro‑inflammatory cytokines (CCL2, TNF‑α, IL‑6) and M1 polarization markers (IL‑6, iNOS, IL‑12), but also abrogated SKF‑mediated suppression of these mediators. Moreover, supplementation with exogenous recombinant IL‑10 (rIL‑10) rescued the detrimental effects of IL‑10 deletion in the cells, both in the presence and absence of SKF (Fig. 6E, F; Supplementary Fig. 10G, H). Notably, IL-10 deficiency exacerbated LPS-induced mitochondrial dysfunction manifested as decreased fluorescence intensity of MitoTracker and amplified intensity of MitoSOX, under which SKF failed to rescue bioenergetic competence or attenuate oxidative stress (Fig. 6G, H; Supplementary Fig. 10H, J). Seahorse analysis confirmed profound OXPHOS collapse in *Il10*^−/−^ BMDMs, represented by impaired basal respiration, reduced maximal respiratory capacity and compromised ATP synthesis. Strikingly, neither LPS nor SKF treatment could further suppressed OXPHOS in *Il10*^−/−^ systems, indicating pre-existing metabolic failure (Fig. 6I). Compared to Fig. 4J, LPS stimulation in WT BMDMs reduced respiration capacity and ATP turnover without significantly affecting basal respiration. While the specific parameters impacted varied between independent experiments, likely due to differences in the animal cohorts or reagent batches, both datasets consistently demonstrated that LPS impaired overall oxidative phosphorylation, indicating compromised mitochondrial bioenergetics. A similar trend was also observed in the expression of key regulators governing OXPHOS, known as AMPK and PGC-1α (Fig. 6J). These findings align with established roles of IL-10 in macrophage mitochondrial modulation [38]. Crucially, the ablation of SKF-mediated bioenergetic control in *Il10*^−/−^ BMDMs defined IL-10 as the terminal effector executing DA-JAML signaling.

**Fig. 7:**
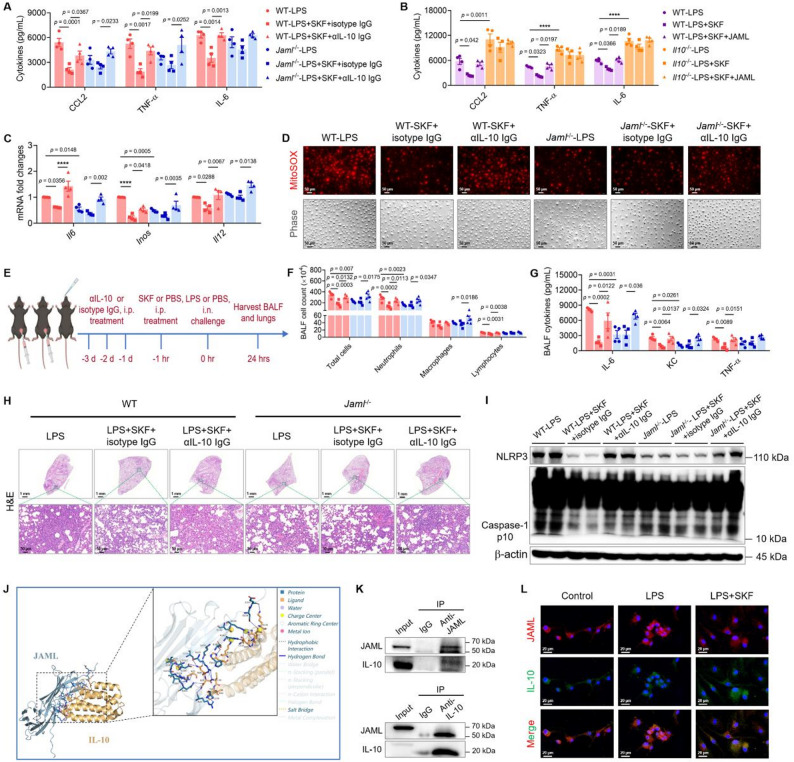
SKF exerts anti-inflammatory actions by reducing JAML expression and its interaction with IL-10 protein. **A-C** BMDMs from WT and *Jaml*^−/−^ mice were treated with SKF (50 µM) combined with isotype IgG (400 ng/mL), SKF combined with αIL-10 IgG (400 ng/mL), or an equal volume of DMSO-containing medium 1 h prior to LPS (100 ng/mL) stimulation (**A**,** C**). BMDMs from WT and *Il10*^−/−^ mice were treated with SKF (50 µM), SKF combined with exogenous JAML protein (1 µg/mL), or an equal volume of DMSO-containing medium 1 h prior to LPS (100 ng/mL) stimulation (**B**). The effects of JAML-IL-10 on the secretion of pro-inflammatory cytokines CCL2, TNF-α, IL-6 (**A**,** B**, *n* = 4 in each group), and the expression of M1 markers *Il6*, *Inos*, *Il12* (**C**, *n* = 4 in each group) by BMDMs stimulated with LPS. **D** Representative images of mitochondrial ROS staining with MitoSOX probe, shown in red, and their corresponding brightfield pictures. Scale bar: 50 μm. *n* = 4 in each group. **E** Illustration of IL-10 depletion using neutralizing antibodies in ALI mice. Specifically, αIL-10 or its isotype control (350 µg/day per mouse intraperitoneally) was administered once daily for three consecutive days before LPS exposure. **F**,** G** Count of total cell number, neutrophil, macrophage, lymphocyte (**F**, *n* = 5 in each group), and generation of cytokines IL-6, KC and TNF-α (**G**, *n* = 4 in each group) in BALF of mice from distinct groups. **H** Representative images of H&E-stained lung tissues at 24 h after LPS (*n* = 5 in each group). Scale bar: 1 mm in the upper panel, 50 μm in the below panel. **I** The activation of pro-inflammatory NLRP3/Caspase-1 pathway in lung tissues of different mice. **J** The optimal binding conformation of JAML and IL-10 proteins as predicted by the algorithm. **K** Reciprocal Co-IP analysis of JAML and IL-10 in BMDMs. **L** Immunofluorescence staining showing the co-localization of JAML and IL-10 in BMDMs. All samples were biologically independent and three or more independent experiments with similar results were performed (“n” represents the number of independent biological replicates). Data are presented as mean ± SEM and analyzed with a 95% confidence interval. Statistical analysis was performed via one-way ANOVA followed by Bonferroni’s post hoc test. ****p < 0.0001

To positionally specify the JAML-IL-10 hierarchy within DA signaling-mediated cyto-protection, we perturbed IL-10 in *Jaml*^−/−^ macrophages while modulated JAML in *Il10*^−/−^ systems, respectively. As seen in Fig. 7A, B and Supplementary Fig. 11A, B, both IL-10 neutralization and JAML addback abolished SKF-mediated suppression of inflammatory cytokine secretion in WT BMDMs. In *Jaml*^⁻/⁻^ macrophages, IL-10 blockade restored CCL2 and TNF-α production even under LPS + SKF treatment. Conversely, in *Il10*^−/−^ macrophages, adding recombinant JAML failed to elevate these cytokines. This demonstrated that JAML acted upstream of IL-10 to mediate the anti-inflammatory effects of SKF in macrophages. Similar trends were also observed in the expression of M1 polarization markers IL-6, iNOS, IL-12, and mROS production, reinforcing the credibility of the aforementioned conclusion (Fig. 7C, D; Supplementary Fig. 11C). We next investigated the effect of DA-JAML-IL-10 axis on the LPS-induced ALI mouse model (Fig. 7E). In line with the in vitro data, JAML deficiency abrogated the protective effects of SKF in ALI, whereas IL-10 neutralizing antibody (αIL-10) significantly increased BALF cellularity and chemokine/cytokine production, as well as exacerbated pathological damage and inflammatory response in lung tissues, an effect independent of JAML status (Fig. 7F-I; Supplementary Fig. 11D-F). These data established IL-10 as the critical downstream effector of the SKF-JAML axis, whereby SKF suppressed JAML expression to upregulate IL-10 protein, which in turn maintained macrophage mitochondrial activity and attenuated uncontrolled inflammation.

The structural features of JAML suggested its capacity to bind intracellular proteins for downstream signal transduction. Given that SKF-JAML-mediated regulation of IL-10 occurred at the protein level rather than the mRNA level (Fig. 6A, B), we hypothesized that SKF may exert its bioactivity by modulating protein-protein interactions between JAML and IL-10. Accordingly, we first demonstrated the binding of these two proteins in macrophages. Computational docking predicted high-affinity binding of JAML-IL-10 (Docking Score = -282.76 kcal/mol) with a confidence score larger than 0.9 (Fig. 7J), which was experimentally validated by reciprocal co-immunoprecipitation (Co-IP) (Fig. 7K). Immunofluorescence analysis further revealed significant co-localization of JAML and IL-10 in BMDMs, and showed that SKF inhibited JAML expression with a consequent increase in IL-10 levels (Fig. 7L; Supplementary Fig. 12A). To explore the functional consequence of this interaction, we examined the effect of JAML on IL‑10 protein stability using the protein synthesis inhibitor cycloheximide (CHX). JAML deficiency significantly prolonged the half‑life of IL‑10 in BMDMs, and treatment with the proteasome inhibitor MG132 blocked the accumulation of IL‑10 protein in *Jaml*^‑/−^ cells, indicating that JAML may promote IL‑10 degradation through the ubiquitin‑proteasome pathway (Supplementary Fig. 12B, C). Collectively, these results proposed a tripartite regulatory module: SKF suppressed JAML transcription and reduced its binding to IL-10 to upregulate bioactive IL-10, thereby orchestrating mitochondrial homeostasis and inflammation resolution in macrophages under LPS challenge.

### Define the bioactivity of DA signaling in human PBMC-derived macrophages from healthy volunteers and ARDS patients

**Fig. 8:**
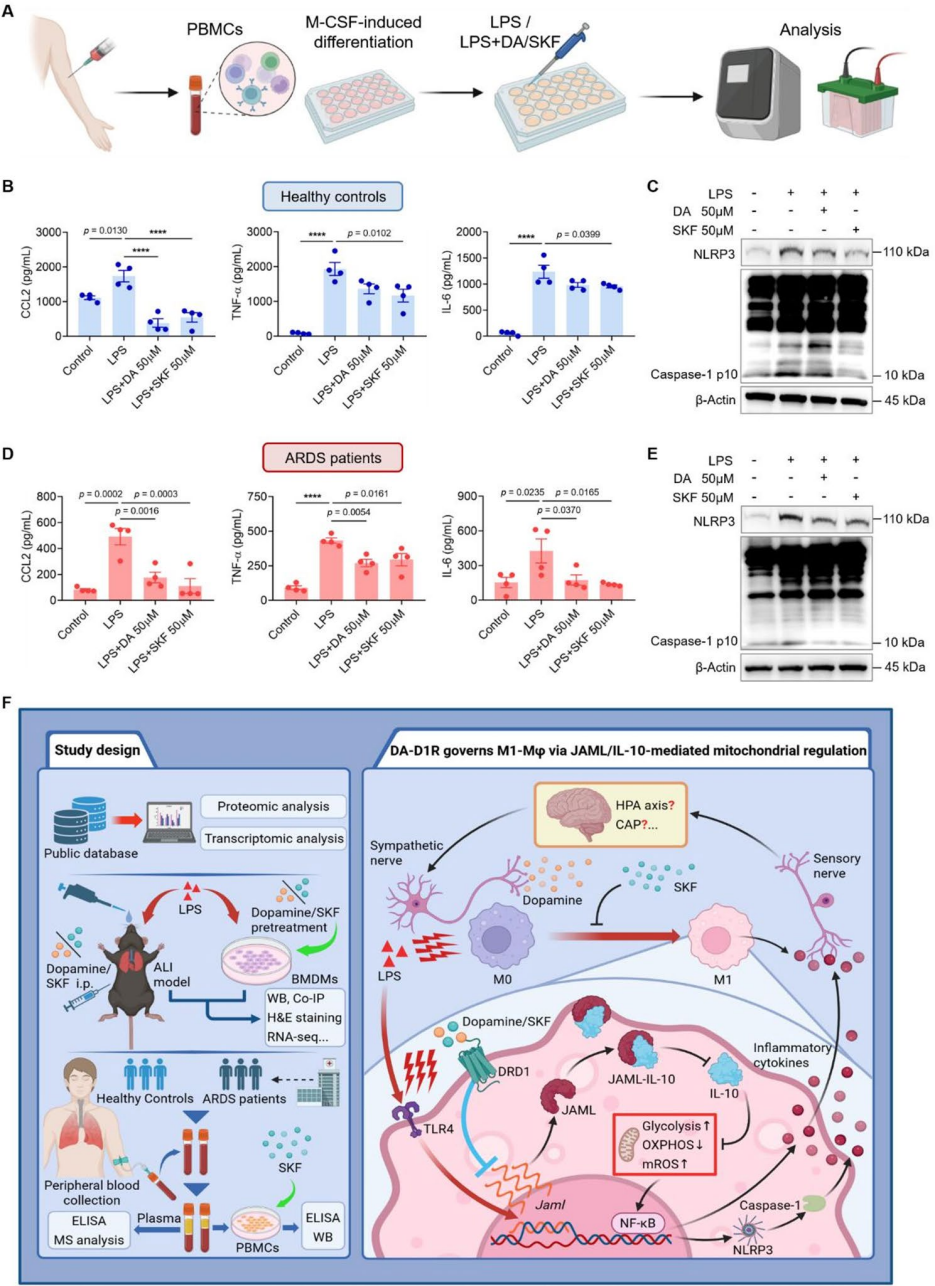
SKF modulates the inflammatory response of human PBMC-derived macrophages. **A** Schematic diagram for PBMC collection, culture, differentiation, and processing. For treatments, BMDMs were treated with DA (50 µM), SKF (50 µM), or an equal volume of DMSO in medium 1 h prior to stimulation with LPS (100 ng/mL) or equal volume of medium. **B**,** C** The effects of SKF on the production of pro-inflammatory cytokines CCL2, TNF-α, IL-6 (**B**, *n* = 4 in each group), and the activation of NLRP3/Caspase-1 signaling pathway (**C**, *n* = 3 in each group) in PBMCs from healthy controls under LPS stimulation. **D**,** E** The effects of SKF on the production of pro-inflammatory cytokines CCL2, TNF-α, IL-6 (**D**, *n* = 4 in each group), and the activation of NLRP3/Caspase-1 signaling pathway (**E**, *n* = 3 in each group) in PBMCs from ARDS patients under LPS stimulation. **F** Illustration of the anti-inflammatory mechanisms of the dopaminergic signaling in macrophages upon LPS challenge. All samples were biologically independent and three or more independent experiments with similar results were performed (“n” represents the number of independent biological replicates). Data are presented as mean ± SEM and analyzed with a 95% confidence interval. Statistical analysis was performed via one-way ANOVA followed by Bonferroni’s post hoc test. ****p < 0.0001. Abbreviations: BMDM, bone marrow-derived macrophage; PBMC, peripheral blood mononuclear cell; WB, western blot; H&E, hematoxylin and eosin; RNA-seq, RNA sequencing; ELISA, enzyme-linked immunosorbent assay; Co-IP, co-immunoprecipitation; MS, mass spectrometry; D1R, D1-like receptor; DRD1, dopamine receptor D1; M0, resting macrophage; M1, classically activated macrophage; HPA, hypothalamic-pituitary-adrenal; CAP, cholinergic anti-inflammatory pathway; TLR4, toll-like receptor 4; JAML, junctional adhesion molecule-like protein; IL-10, interleukin-10; NF-κB, nuclear factor kappa-light-chain-enhancer of activated B cells; NLRP3, NOD-like receptor family pyrin domain containing 3; OXPHOS, oxidative phosphorylation; mROS, mitochondrial reactive oxygen species

To assess the clinical translational potential of DA signaling, human PBMCs were isolated from both ARDS patients and healthy controls and differentiated into macrophages (*n* = 4 each group) (Fig. 8A). Baseline characteristics showed no significant intergroup differences (Table S1). First, we confirmed that SKF/DA with or without LPS treatment did not have toxicity to the cells up to 50 µM for 24 h (Supplementary Fig. 13). In PBMCs from healthy volunteers, we found that SKF suppressed the secretion of pro‑inflammatory chemokines and cytokines typically induced by LPS, including CCL2, TNF‑α, and IL‑6, while DA only significantly inhibited CCL2 secretion (Fig. 8B). Both agents markedly attenuated LPS‑triggered activation of the NLRP3/Caspase‑1 pathway (Fig. 8C). By contrast, PBMCs from ARDS patients exhibited reduced cytokine production upon LPS challenge compared with healthy controls, suggesting an impaired immune responsiveness that may represent a protective mechanism against excessive inflammation upon continuous or repeated endotoxin exposure [39–41]. In this setting, both SKF and DA retained significant modulatory effects, characterized by reduced secretion of pro‑inflammatory cytokines and suppression of inflammatory pathway activation (Fig. 8D-E). These data indicate that dopaminergic signaling exerts conserved immunomodulatory activity in both healthy individuals and critically ill patients, with DA and SKF showing comparable efficacy in the latter group.

## Discussion

In recent years, efforts have been directed toward intervening in pulmonary inflammation in ALI by targeting neuroimmune interactions, including the cholinergic anti-inflammatory pathway, sympathetic-immune axis, purinergic signaling, neuropeptide-mediated pathways, and the renin-angiotensin system [6]. Clinical trials have demonstrated that routine administration of the β₂AR agonist albuterol failed to improve outcomes in mechanically ventilated ALI patients (NCT 00434993) [42, 43]. Similarly, salmeterol did not prevent ALI onset or ameliorate organ failure, survival, or health-related quality of life in post-esophagectomy patients (ISRCTN47481946) [44, 45]. The β₂AR activation by Gs protein and guanosine triphosphate leads to cyclic adenosine monophosphate (cAMP) production. This cascade further activates protein kinase A and transcription of the cAMP response element-binding protein, thereby triggering the expression of multiple anti-inflammatory genes [46]. This pathway parallels the canonical signaling activated by dopamine D1 receptors in certain cell and animal models, though notably, it is not engaged in human monocyte-derived macrophages [47]. However, the autoregulatory desensitization of β₂AR limits cAMP-dependent anti-inflammatory signaling [48], which is a key factor underlying the suboptimal results observed in clinical trials of β₂AR agonists. A deeper understanding of the mechanistic basis of neuroimmune crosstalk in ALI is essential to accelerate the clinical translation of these therapeutic strategies. In this study, we investigated the role of pulmonary dopaminergic signaling in LPS-induced acute lung inflammation and elucidated a cAMP-independent anti-inflammatory mechanism mediated by DA and the D1R agonist SKF. Mechanistically, SKF suppressed *Jaml* transcription, leading to reduced JAML protein level. Diminished JAML binding to IL-10 therefore orchestrated mitochondrial metabolic reprogramming and polarization in macrophages, ultimately yielding potent anti-inflammatory effects (Fig. 8F).

To elucidate the regulatory mechanism of the dopaminergic signaling in ALI, a critical gap must be addressed: how the immune system modulates neuronal function through inflammatory mediators. Specifically, the impact of immune cell-derived cytokines/chemokines on dopaminergic neurons during ALI remains poorly characterized, with limited studies available. Our analysis of multiple public databases revealed enhanced DA synthesis and metabolism during LPS-induced acute inflammation (Fig. 1A-D). Mass spectrometry of peripheral blood from ARDS patients and lung tissue from ALI mice showed decreased levels of DA and norepinephrine in the acute inflammatory phase, while their respective major metabolic end products, HVA and VMA were significantly elevated (Fig. 1E, G). Furthermore, expression of D1Rs and the DA-synthesizing core key enzymes TH and DDC was reduced in ALI mouse lung tissue during the peak inflammatory phase (Fig. 1H-L). Together, these findings suggest that DA is rapidly synthesized and consumed during ALI/ARDS progression, indicating its potential involvement in pulmonary immunomodulation. We hypothesized that this inflammatory signal, along with the resultant dopaminergic deficit, was transmitted to the brain via sensory afferent pathways. Subsequently, efferent autonomic signaling might enhance peripheral dopaminergic tone to restore homeostasis (Fig. 8F). However, functional validation of this integrated neuroimmune circuit warrants further investigation.

Several studies have reported seemingly contradictory roles for DA in regulating inflammation in macrophages. On one hand, activation of D1Rs has been shown to inhibit NLRP3 inflammasome activation, potentially through mechanisms involving NLRP3 ubiquitination and degradation or the cAMP signaling pathway [14, 15]. Our study reveals an additional mechanism: metabolically-driven suppression of both NF-κB and inflammasome activation upon D1R stimulation. This may explain why D1R activation (20 µM DA) in human monocyte-derived macrophages, which notably lacks a functional cAMP-protein kinase A pathway [49], can still exert anti-inflammatory effects under LPS challenge [50]. On the other hand, some reports indicate that DA can enhance inflammatory responses. For instance, in human macrophages, a lower concentration of DA (1 µM) has been shown to activate NF-κB signaling and promote inflammasome formation [51], or increase the production of inflammatory factors [52]. This apparent discrepancy may be explained by several factors. First, the anti-inflammatory effect of DA appears to require a threshold concentration (likely > 10 µM), particularly in the presence of an inflammatory stimulus like LPS. Second, the pro- and anti-inflammatory effects may be mediated by distinct signaling pathways and potentially different DR subtypes. The absence of functional cAMP-PKA signaling in human macrophages further suggests that the anti-inflammatory effect observed under LPS challenge is mediated through alternative pathways. Our study demonstrates that inhibition of JAML expression and elevation of IL-10 levels represent one such mechanism through which D1R activation suppresses NF-κB and NLRP3 inflammasome signaling. While we recognize that no single pathway can fully account for the complexity of dopaminergic immunomodulation, our work provides a novel mechanistic branch of this regulatory network.

Our preliminary study identified a key target of SKF: the activation of the nuclear factor erythroid 2-related factor 2 antioxidant system [22]. This finding suggests that dopaminergic signaling enhances the intrinsic anti-inflammatory capacity of macrophages during acute inflammation. This logically directed our focus to mitochondrial energy metabolism, a core regulator of cellular defense and homeostasis. While the role of DA signaling in immunometabolic reprogramming during ALI remained unclear, our study now demonstrated that SKF treatment reprogramed mitochondrial metabolism to favor OXPHOS over glycolysis, thereby ameliorating LPS-induced mitochondrial dysfunction (Fig. 4). Specifically, SKF enhanced OXPHOS while inhibited glycolysis under LPS stimulation, thereby restricting excessive polarization toward the pro-inflammatory M1 phenotype. This work established a concept of a neuroimmune-metabolic circuit orchestrated by the dopaminergic signaling in ALI, representing a new regulatory layer in our understanding of its pathogenesis. Interestingly, D1R activation failed to modulate M2 polarization, and the possible reasons are speculated as follows. As a transmembrane glycoprotein, the interaction of JAML with intracellular proteins may depend on the specific signaling environment of M1 activation, such as TLR4 activation induced by LPS. M2 polarization is usually driven by factors like IL-4/IL-13 and relies on the STAT6 pathway. In this context, JAML may not be effectively activated or bind to downstream molecules, so changes in its expression do not affect the M2 phenotype. In addition, M2-type macrophages prefer metabolic modes of OXPHOS and fatty acid oxidation [53, 54], while SKF could not increase the level of fatty acid oxidation in BMDMs (data not shown), making the DA/D1R/JAML pathway a “precision regulatory axis” targeting M1 polarization.

Through RNA-seq analysis on BMDMs, we identified JAML as the top-ranked molecule modulated by SKF (Fig. 5A). Discovered in 2003, JAML was a new member of the junctional adhesion molecules superfamily and belonged to the secretory type I transmembrane glycoproteins [37]. The structure of JAML, from the amino terminus to the carboxyl terminus, mainly consisted of two extracellular V-set immunoglobulin domains, a stalk region that connected the extracellular domain to the cell membrane, a transmembrane helix domain, and an intracellular domain. During acute inflammation, the extracellular region could be cleaved at the stalk and shed from the cell membrane, subsequently functioning as a secretory protein [33]. The intracellular domain, which was highly conserved, mediated signal transduction through interactions with intracellular proteins. Besides, JAML was also expressed in the cytoplasm, and damage stimuli could promote its translocation to the membrane [32]. Previous studies have shown that JAML was primarily present in innate immune cells, such as monocytes/macrophages and neutrophils. Its expression level was significantly upregulated after various stimuli, participating in the regulation of leukocyte migration and activation, and promoting the progression of inflammation [55, 56]. In recent years, scholars have begun to explore its new immunometabolic mechanisms. In diabetic kidney disease, JAML promoted lipid deposition in podocytes by regulating their lipid metabolism, thereby exacerbating glomerular damage [57]. Similarly, our study indicated that JAML bridged the dopaminergic signaling and mitochondrial metabolism by affecting the protein level of IL-10 in macrophages, and further regulated downstream inflammatory responses (Figs. 6 and 7). The intimate interplay between JAML and cellular metabolism transcends its typical adhesive properties, making it a key orchestrator in governing tissue injury and metabolic rewiring. The structural architecture of JAML implicated intracellular protein binding as a primary mechanism for its signal transduction activity. This study further established that JAML directly suppressed IL-10 expression via protein-protein interaction. Immunofluorescence and Co-IP assays confirmed JAML-IL-10 complex formation, which reduced IL-10 bioavailability (Figs. 6A and B and 7K and L). Mechanistically, we demonstrated that JAML might promote IL‑10 degradation via the ubiquitin‑proteasome pathway, as JAML deficiency prolonged IL‑10 protein half‑life and the proteasome inhibitor MG132 reversed IL‑10 accumulation in *Jaml*^‑/−^ cells (Supplementary Fig. 12). Whether additional mechanisms, such as lysosome‑dependent degradation or conformational destabilization of IL‑10, also contribute to this regulation remains to be explored. Overall, these findings highlight the critical role of JAML in ALI-related pulmonary inflammation, especially considering the current paucity of research on JAML in the context of ALI.

While this study provides preliminary insights into the regulatory role of dopaminergic signaling in ALI, several limitations warrant further in-depth investigation. First, the sensory nerve circuits activated by pulmonary inflammation, including specific nerve subtypes, central processing regions, and pulmonary dopaminergic neuron populations, remain uncharacterized. Second, the clinical relevance of our findings is constrained by the relatively small patient cohort and the observational study design. The limited sample size may affect the generalizability of the results, and confounding by indication must be acknowledged, patients who received DA tended to present with more severe circulatory instability, a factor independently linked to worse outcomes. Therefore, whether SKF or DA can meaningfully improve clinical outcomes in ALI/ARDS patients requires prospective validation in larger, controlled studies. Besides, while our pharmacological controls strongly support a D1-receptor-mediated mechanism, the use of high concentrations of SKF does not entirely preclude the possibility of weak off-target binding. We state that future studies would benefit from employing newer, more selective D1-receptor agonists (e.g., A-68930) and in vitro binding assays to further confirm absolute specificity. In addition, future studies will generate macrophage-specific conditional knockout mice for JAML and IL-10 to more precisely validate the conclusions of this study. In the present study, we identified the potential regulatory effects of DA secreted by macrophages; however, future studies are needed to directly measure endogenous dopamine in this context and to employ genetic approaches to confirm this autocrine loop.

## Conclusion

This study explored the neuroimmune regulation of lung inflammation in ALI by integrating public database mining, clinical cohorts, and animal models, revealing an undescribed protective axis. The computational and experimental analyses suggested accelerated DA synthesis and consumption during acute pulmonary inflammation. Then we demonstrated that dopaminergic signaling conferred anti-inflammatory protection via D1R activation in macrophages, specifically through SKF-mediated maintenance of mitochondrial homeostasis and regulation of inflammatory responses via the JAML/IL-10 axis. Mechanistically, the DA-D1R signaling sustained mitochondrial fitness, enhanced OXPHOS, and inhibited glycolysis through the interaction between JAML and IL-10 proteins, thereby preventing excessive macrophage polarization toward the pro-inflammatory M1 phenotype and ultimately alleviating inflammatory damage in ALI. This work thus elucidated the dopaminergic signaling as a pivotal nexus linking neuroimmune-metabolic crosstalk in ALI, transcending mere pathway characterization to offer a novel paradigm for understanding complex inflammatory disorders and paving the way for targeted therapeutic strategies that harness neuro-regulation to restore immune-metabolic homeostasis.

## Methods

### Study approval

All animal experiments were performed in accordance with the Guide for the Care and Use of Laboratory Animals issued by the Shanghai Laboratory Animal Management Committee. Additionally, this research protocol was approved by the Ethics Review Committee of the Experimental Animal Research Center of Tongji University (License number: TJBB03723103, Shanghai, China). All procedures were conducted under sodium pentobarbital anesthesia, with every effort made to minimize animal suffering and reduce the number of animals used.

The collection of peripheral blood and clinical information from ARDS patients and healthy volunteers was approved by the Ethics Committee of Shanghai East Hospital, China (Approval number: 2019YYS138, Shanghai, China), and all participants provided written informed consent.

### Establishment of LPS-induced ALI mouse model

The control (C57BL/6) mice, wild type (WT) mice, B6.129P2-*Il10*^tm1/Nju^ (*Il10*^−/−^) mice, and C57BL/6Smoc-*Jaml*^em1Smoc^ (*Jaml*^−/−^) mice (both male and female, 6–8 weeks old) were purchased from Shanghai Model Organisms Center, Inc. (Shanghai, China) and housed in a specific pathogen-free environment. They were maintained under a standard 12-hour light/dark cycle with free access to food and water. Mice were treated via intranasal instillation of LPS (10 mg/kg; Sigma-Aldrich, USA) or an equal volume of sterile phosphate-buffered saline (PBS, control group). Following anesthesia by intraperitoneal injection of sodium pentobarbital (10 mg/mL, Yiheng, China) at a dose of 50 mg/kg, LPS or PBS was slowly instilled into the nasal cavity in 2–3 aliquots to ensure inhalation into the lungs. Mice were sacrificed at 0, 2, 6, 12, and 24 h, or 4 and 7 days post-challenge for subsequent analyses.

Other drug intervention protocols were as follows: DA (300 µg per mouse intraperitoneally; Sigma-Aldrich, USA), SKF (5 mg/kg intraperitoneally; MCE, USA), or Cab (5 mg/kg intraperitoneally; MCE, USA) were given 1 h, and SCH (0.5 mg/kg intraperitoneally; MCE, USA) was administered 2 h prior to LPS stimulation; MPTP was delivered intranasally 4 times at 2-h intervals (15 mg/kg per administration; Sigma-Aldrich, USA) before LPS challenge; exogenous JAML (100 µg/kg; MCE, USA) was intranasally instilled concurrently with LPS; Clo-lip or PBS-lip (5 mg/mL, 75 µL per mouse intratracheally; Liposoma BV, the Netherlands) were given 73 h before LPS; αIL-10 or its isotype control (350 µg/day per mouse intraperitoneally; BioXcell, USA) was administered once daily for three consecutive days prior to LPS exposure. Randomization and blinding were employed to minimize bias.

### Extraction and processing of BALF

Mice were anesthetized via intraperitoneal injection of sodium pentobarbital. Subsequently, 0.8 mL of ice-cold PBS was slowly injected into the trachea and carefully aspirated, with the recovered volume exceeding 70% of the injected volume. BALF samples were centrifuged at 140×g for 10 min at 4 °C to harvest bronchoalveolar inflammatory cells. Total cell counts were determined using the trypan blue exclusion assay with a hemocytometer. Following cytocentrifugation, BALF samples were fixed on glass slides, stained with Wright-Giemsa (Baso Diagnostics, China), and differential cell counting was performed by blindly counting at least 200 cells under a microscope at 400× magnification. The fields for differential cell counting were selected randomly across the slide to ensure unbiased and objective quantification. The BALF supernatant was then aliquoted and stored at − 80 °C for subsequent experimental analyses. To mitigate protein degradation, we implemented a stringent protocol. Specifically, immediately after collection, all BALF samples were kept on ice continuously. The processing pipeline was rigorously standardized: samples were centrifuged within 30 min of lavage, supernatant was aliquoted into minimal single-use volumes (50 µL/tube) to avoid freeze-thaw cycles, and flash-frozen at -80 °C until analysis. Thawed aliquots were used once and discarded.

### Histoimmunological profiling of pulmonary injury and neutrophil activation

For histological analysis, the left lung lobe was excised without prior BALF collection. The harvested tissue was fixed in 4% neutral formaldehyde, embedded in paraffin, and sectioned into 5-µm-thick slices, which were mounted on glass slides. Hematoxylin and eosin (H&E) staining was then performed, followed by dehydration and coverslipping. Lung injury was evaluated by two independent investigators blinded to sample information using a validated scoring system [58]. To assess damage severity, six randomly selected fields were analyzed based on five parameters: alveolar space neutrophils, interstitial neutrophils, hyaline membranes, proteinaceous debris in airspaces, and alveolar septal thickening. Each parameter was scored 0, 1, or 2 according to severity, with weights assigned based on their relevance. Weighted scores for all parameters were averaged across the evaluated fields, yielding a final score ranging from 0 to 1. Slides were also immunostained with anti-Ly6G (Abcam, ab25377, 1:100, UK) antibody, followed by FITC-goat anti-rat IgG secondary antibody (Servicebio, GB22302; 1:100, China) to assess neutrophil accumulation and activation status. Images were captured using a Nikon Eclipse C1 microscope (Nikon, Japan).

### Isolation and treatment of mouse BMDMs

Six- to eight-week-old mice were sacrificed via carbon dioxide exposure. Tibiae and femurs were then harvested, cut, and the bone marrow cavities were flushed with PBS using a 5 mL syringe, collecting the marrow into centrifuge tubes. The resulting bone marrow cell suspension was centrifuged at 220×g for 5 minutes at room temperature to pellet the cells. Cells were resuspended in Dulbecco’s Modified Eagle Medium (DMEM; Gibco, USA) supplemented with 1% penicillin-streptomycin (Gibco, USA), 10% heat-inactivated fetal bovine serum (Gibco, USA), and 20 ng/mL macrophage colony-stimulating factor (PeproTech, USA). On day 4 of culture, fresh complete medium (equivalent to half the initial volume) was added. On day 7, the medium and non-adherent cells were removed, and adherent BMDMs were detached using cell dissociation solution [59]. After collection and counting, cells were seeded into 96-well plates (5 × 10^4^ cells/well), 12-well plates (5 × 10^5^ cells/well), or 6-well plates (1 × 10^6^ cells/well) for subsequent experiments. The specific drug intervention protocols were as follows: DA (0.05 µM, 0.5 µM, 5 µM, 50 µM; Sigma-Aldrich, USA), SKF (0.1 µM, 1 µM, 10 µM, 50 µM; MCE, USA), Cab (0.1 µM, 1 µM, 10 µM, 50 µM; MCE, USA), SCH (1 µM, 10 µM, 20 µM, 50 µM; MCE, USA), exogenous JAML protein (1 µg/mL; MCE, USA), rIL-10 (100 ng/mL; PeproTech, USA), αIL-10 IgG (400 ng/mL; BioXcell, USA), as well as their corresponding controls were administered 1 h prior to stimulation with LPS (100 ng/mL; InvivoGen, France) or IL-4/IL-13 (20 ng/mL; Novoprotein, China). After co-cultured with the aforementioned drugs for different time points, BMDMs or the supernatants were collected for further detection. To assess IL-10 protein stability, BMDMs from WT and *Jaml*^−/−^ mice were treated with CHX (20 µg/mL; MCE, USA) and harvested at indicated time points (0, 1, 4, and 8 h). For proteasome inhibition experiments, BMDMs were treated with MG132 (10 µM; MCE, USA) and collected at 0 and 6 h after treatment. Whole-cell lysates were then prepared and analyzed by immunoblotting.

### Processing of human blood samples and differentiation of macrophages

Ten milliliters of venous blood from ARDS patients or healthy volunteers were drawn into an EDTA anticoagulant tube, which was gently inverted to mix thoroughly and prevent coagulation. The blood was mixed with an equal volume of PBS by gentle pipetting up and down to homogenize. A 5-mL aliquot of Ficoll separation medium (GE Healthcare, USA) was added to a centrifuge tube, and the diluted blood was slowly overlaid onto the medium surface to form a distinct layered interface. Centrifugation was performed at 400×g for 20 min at room temperature with the centrifuge brake disabled. After centrifugation, the PBMC layer was carefully aspirated using a pipette and transferred to a new centrifuge tube. Five volumes of PBS were added, and the tube was gently inverted to mix. The mixture was centrifuged at 300×g for 10 min at room temperature, and the supernatant was discarded. This washing step was repeated 1–2 times. Freshly isolated PBMCs were resuspended in complete culture medium (RPMI-1640 with 10% heat-inactivated fetal bovine serum and 1% penicillin-streptomycin). Cells were seeded at a density of 1 × 10⁶ cells/mL in 12-well plates and allowed to adhere for 24 h. Following this adherence period, non-adherent cells were removed by gentle washing, and the remaining adherent monocyte-derived macrophages were cultured in fresh complete medium containing 10 ng/mL human macrophage colony-stimulating factor (PeproTech, USA). The medium was refreshed on day 3, and cells were considered mature macrophages after 7 days. Subsequently, the differentiated cells were treated with DA or SKF (50 µM) for 1 h prior to stimulation with LPS (100 ng/mL). Cells and supernatants were harvested 24 h post-LPS stimulation for subsequent analysis.

### Neurotransmitters and their metabolites detection

Human peripheral blood samples were centrifuged at 1200×g for 10 min at 4 °C within 30 min of collection. The resulting plasma supernatant or freshly harvested mouse lung tissue, was then aliquoted. To prevent analyte degradation, each aliquot was flushed with nitrogen gas, sealed, and stored at -80 °C until analysis. Neurotransmitters (dopamine, noradrenaline) and their metabolites (homovanillic acid (HVA), vanillylmandelic acid (VMA)) were detected and quantified using ultra-performance liquid chromatography electrospray ionization-tandem mass spectrometry (UPLC-ESI-MS/MS) on a Waters I-Class UPLC system coupled to an AB Sciex Qtrap 6500 + triple quadrupole mass spectrometer.

### Cell viability assay

Mouse BMDMs or human PBMCs were seeded in 96-well plates at a density of 5 × 10^4^ or 1 × 10^5^ cells/well. The cell viability assays for both BMDMs and PBMCs were consistently performed 24 h after the final drug administration via CCK-8 assay (Dojindo Laboratories, Japan). The working reagent, diluted 10-fold in culture medium, was added. After incubation for 1–2 h, the absorbance of each well was measured at 450 nm using a microplate reader (SpectraMax iD3, Molecular Devices, Sunnyvale, USA). Cell viability (%) was calculated as follows: [(Absorbance of sample well - Absorbance of blank well) / (Absorbance of control well − Absorbance of blank well)] × 100%.

### Immunoblotting assay

Following distinct treatments, cells or homogenized lung tissues were lysed in RIPA lysis buffer (Beyotime, China). Extracted proteins were quantified using a BCA protein assay kit (Beyotime, China) and denatured by boiling at 95 °C for 10 min. Equivalent amounts of protein samples were loaded onto 10% (w/v) sodium dodecyl sulfate-polyacrylamide gels, electrophoresed, and transferred to 0.45-µm PVDF membranes (Millipore, USA). Membranes were then blocked with 5% (w/v) nonfat milk in Tris-buffered saline (TBS) containing 0.1% (v/v) Tween 20 (TBST) for 1.5 h at room temperature. Membranes were then washed in Tris-buffered saline with Tween 20 (TBST), blocked with 5% (w/v) skim milk for 1.5 h at room temperature. Each membrane strip was washed and then incubated with specific primary antibodies targeting β-actin (Cell Signaling Technology, 4970, 1:1000, USA), DRD1 (Abcam, ab256555, 1:1000, UK), DRD5 (Abcam, ab181623, 1:1000, UK), DDC (Cell Signaling Technology, 13561, 1:1000, USA), DRD2 (Proteintech, 55084-1-AP, 1:1000, USA), DRD3 (Abcam, ab155098, 1:1000, UK), DRD4 (Proteintech, 28094-1-AP, 1:1000, USA), TH (Zen-Bioscience, R381111, 1:1000, China), NLRP3 (Cell Signaling Technology, 15101, 1:1000, USA), Caspase-1 p10 (Abcam, ab179515, 1:1000, UK), Caspase-1 p20 (Cell Signaling technology, 89332, 1:1000, USA), IL-1β (Cell Signaling technology, 12242, 1:1000, USA), GSDMD (Cell Signaling technology, 10137, 1:1000, USA), TLR4 (Proteintech, 66350-1-Ig, 1:1000, USA), p-P65 (Cell Signaling technology, 3033, 1:1000, USA ), iNOS (Abcam, ab178945,1:1000, UK), ARG1 (Cell Signaling technology, 93668, 1:1000, USA), OPA1 (Cell Signaling Technology, 80471, 1:1000, USA), DRP1 (Cell Signaling Technology, 8570, 1:1000, USA), p-DRP1 (Cell Signaling Technology, 4494, 1:1000, USA), Hif-1α (Cell Signaling technology, 14179, 1:1000, USA), GLUT (Cell Signaling technology, 12939, 1:1000, USA), p-AMPK (Cell Signaling Technology, 2535, 1:1000, USA), JAML (Abcam, ab183714, 1:1000, UK), and IL-10 (BIOSS, bs-0698R,1:1000, China) at 4 °C overnight. The following day, membranes were washed 3 times in TBST, incubated with horseradish peroxidase-conjugated anti-rabbit or anti-mouse secondary antibodies (Cell Signaling Technology, 7074, 7076, 1:2000, USA) for 1.5 h, and washed again. The bands were visualized using a multi-functional chemiluminescence imaging system (Tanon 5200, Tanon Biotechnology, China), and images were analyzed by ImageJ software. For quantitative analysis, the expression of each target protein was normalized to the β-actin signal from its own lane. For the detection of proteins with the same molecular weight on already developed blots, we used a rapid stripping buffer (Epizyme, China) to strip the membranes for 5 min. After washing with 1×TBST for 5 min, the membranes were blocked in 5% (w/v) skim milk at room temperature for 1.5 h, followed by re-incubation with primary and secondary antibodies and subsequent development as described above. The uncropped immunoblot images corresponding to Figs. 1H, 3N, 4C, I and N, 5E and N, 6B and D and 7I and K, and Fig. 8C, E were supplied in Supplementary Figs. 14 and 15.

To ensure specificity for DRD1 and DRD5, we used rigorously validated antibodies (ab256555 for DRD1; ab181623 for DRD5, targeting a unique C-terminal peptide). Our confidence stems from their specific immunogen design, validation data provided by the supplier, and their clear detection of single bands at the expected molecular weights in our assays. Although we did not perform additional peptide-blocking experiments, the cumulative evidence strongly supports their specificity as used in our study. This careful application adds to the practices for employing dopaminergic receptor antibodies in immunoblotting.

### RNA extraction and reverse transcription

The centrifuge was pre-cooled to 4 °C. Culture medium was aspirated from plates, and cells were washed with sterile PBS. An appropriate volume of TRIzol reagent (Invitrogen, USA) was added, and cells were lysed by pipetting, then transferred to an EP tube. Chloroform was added, the mixture was vortexed, and incubated at room temperature for 3 min. After centrifugation at 14,000 rpm for 15 min at 4 °C, the supernatant was aspirated, mixed with isopropanol, and allowed to stand. Subsequent centrifugation yielded an RNA pellet, which was washed with 75% ethanol, centrifuged, and air-dried. The RNA was dissolved in DEPC-treated water, mixed with PrimeScript Reverse Transcriptase reagents (Takara, Japan), and subjected to reverse transcription at 37 °C for 15 min followed by 85 °C for 5 s. cDNA purity was assessed using a spectrophotometer, and samples were diluted to 500 ng/µL.

### Quantitative-polymerase chain reaction (qPCR)

A 10-µL reaction system was prepared with TB Green Premix Ex Taq™ (Takara, Japan) according to the target primers, loaded onto the instrument, and run on the QuantStudio™ 6 Flex Real-Time PCR System. Relative gene expression was calculated using the 2^–∆∆Ct^ method. Specific primers were purchased from BioTNT (China), with sequences listed as follows:

- Mouse IL-6, Forward: TAGTCCTTCCTACCCCAATTTCC,
- Reverse: TTGGTCCTTAGCCACTCCTTC;
- Mouse iNOS, Forward: TGCCACGGACGAGACGGATAG,
- Reverse: CTCTTCAAGCACCTCCAGGAACG;
- Mouse IL-12, Forward: CAGAAAGGTGCGTTCCTCGTA,
- Reverse: GCCCCTTTGCATTGG;
- Mouse ARG1, Forward: CTCCAAGCCAAAGTCCTTAGAG,
- Reverse: AGGAGCTGTCATTAGGGACATC;
- Mouse YM1, Forward: AGAAGGGAGTTTCAAACCTGGT,
- Reverse: GTCTTGCTCATGTGTGTAAGTGA;
- Mouse IL-10, Forward: GCTCTTACTGACTGGCATGAG,
- Reverse: CGCAGCTCTAGGAGCATGTG;
- Mouse AMPK, Forward: AATAGCCCATGAGCTCCAGA,
- Reverse: TGCAGCCCTACACTGAAATG;
- Mouse PPAR, Forward: GCAGCTCGTACAGGTCATCA,
- Reverse: CTCTTCATCCCCAAGCGTAG;
- Mouse PGC-1ɑ, Forward: AAGTGGTGTAGCGACCAATCG,
- Reverse: AATGAGGGCAATCCGTCTTCA;
- Mouse Hif-1ɑ, Forward: TGCTCATCAGTTGCCACTTC,
- Reverse: TGGGCCATTTCTGTGTGTAA;
- Mouse HK2, Forward: GAATGGGAAGTGGGGTGGAG,
- Reverse: TGTGGTCAAAGAGCTCGTC;
- Mouse PFKP, Forward: GGAAGCCAAATGGGACTGT,
- Reverse: CGCACTACCGATGATGGTC;
- Mouse JAML, Forward: CTGCCAGGCTTGACCGTTTC,
- Reverse: CGCTGGACAACACACATCCCAT;
- Mouse β-actin, Forward: AACAGTCCGCCTAGAAGCAC,
- Reverse: CGTTGACATCCGTAAAGACC.

### Cytokine analysis

The levels of IL-6 (Invitrogen, 88-7064-88, USA), KC (MultiSciences Biotech, EK296, China), TNF (Invitrogen, 88-7324-88, USA) in mouse BALF, CCL2 (MultiSciences Biotech, EK287, China), TNF, IL-6 in the culture supernatants of mouse BMDMs, and CCL2 (MultiSciences Biotech, EK187, China), TNF (MultiSciences Biotech, EK182, China), IL-6 (MultiSciences Biotech, EK106, China) in the supernatants of cultured human PBMCs were quantified using ELISA kits according to the manufacturer’s protocols.

### Proteomics data processing

Publicly available proteomics data were retrieved from ProteomeXchange Datasets using the keywords “acute lung injury” and “acute respiratory distress syndrome”. Following standardized data processing, differences in the expression of neural proteins between the control group and the ALI/ARDS group were identified. Data processing and analysis were performed using MaxQuant and the R programming language, with the specific workflow as follows. For label-free data, after importing raw data, database searching was performed using the label-free quantification method with reference to UniProt global parameters. Following acquisition of protein expression abundances across groups, the R programming language was used for missing value removal and subsequent analyses. For labeled data, based on the labeling strategy employed (typically tandem mass tags [TMT] or isobaric tags for relative and absolute quantitation [iTRAQ]), database searching was conducted using the corresponding labeling-specific parameters [60].

### Genomics data processing

Publicly available genomic data were retrieved from GEO datasets using the keywords “acute lung injury” and “acute respiratory distress syndrome”. Following standardized data processing, differences in the expression of neural protein-related genes between the control group and the ALI/ARDS group were identified. All data processing and analyses were performed using the R programming language, with the specific workflow as follows: the target datasets were directly downloaded and extracted using R. Probe IDs were annotated to corresponding genes based on platform information. Neural protein-related genes were then extracted from the annotated expression matrix, exported, and subjected to further analysis.

### Co-immunoprecipitation (Co-IP)

Following intervention, macrophages were lysed with a buffer containing 50 mM Tris, 150 mM NaCl, 1 mM EDTA, and 0.2% Triton X-100, supplemented with a protease inhibitor cocktail. The assay was performed using the Co-IP Kit according to the manufacturer’s instructions (Beyotime, P2179, China). Briefly, lysates were centrifuged at 12,000×g for 15 min at 4 °C, and supernatants were collected as whole-cell lysate samples. Protein G magnetic beads were added to the lysates and incubated overnight at 4 °C. After washing, 10 µg of anti-JAML antibody (Abcam, ab183714, 1:50, UK), anti-IL-10 antibody (BIOSS, bs-0698R,1:50, China), or isotype-matched control IgG was added, followed by incubation at room temperature for 2 h. Magnetic beads bound to the antibody-protein complexes were washed, and loading buffer was added. Co-IP samples were denatured at 95 °C for 5 min in a metal bath, magnetic beads were removed, and the resulting protein samples were subjected to immunoblotting analysis.

### Immunofluorescence and mitochondrial staining

BMDMs were seeded on cell climbing slices and subjected to various stimuli at 37 °C for 24 h. Adherent cells were then fixed with 4% paraformaldehyde for 15 min at room temperature, permeabilized with 0.1% Triton X-100 for 10 min, and blocked with 5% BSA for 1 h. Cells were incubated overnight at 4 °C with primary antibodies against CD68 (Servicebio, GB113109; 1:200, China), CD86 (Proteintech, 13395-1-AP, 1:300, USA), or CD206 (Servicebio, GB113497; 1:500, China). Following PBS washes, slides were incubated for 2 h in the dark with secondary antibodies in blocking buffer containing DAPI (1:1000): goat anti-rabbit IgG-Cy3 (Servicebio, GB21303; 1:300, China) or goat anti-rat IgG-FITC (Servicebio, GB22302; 1:100, China). Sections were mounted with ProLong Antifade reagent (ThermoFisher, USA) and stored overnight at room temperature in the dark. Photos were acquired on a Nikon Eclipse C1 fluorescence microscope (Japan) with DS-U3 imaging system.

For mitochondrial integrity and activity measurement, BMDMs were seeded in 4-well cover glass-bottom chamber and treated with distinct drugs for 24 h. Cells were then washed three times with PBS to remove residual medium, followed by incubation with MitoTracker Red CMXRos (Invitrogen, M7512, USA) to label the mitochondria, MitoSOX Red/Green (Invitrogen, M36008/M36006, USA) to evaluate mitochondrial ROS-scavenging activity, and DCFH-DA (Sigma-Aldrich, D6883, USA) to analyze the generation of cellular total ROS. The cell nuclei were stained with Hoechst (Beyotime, C1017, China) prior to confocal imaging. Images were acquired using confocal microscopy (TCS SP8, Leica, Germany) and processed in ImageJ. For each sample, six non-overlapping regions were randomly chosen. Within each region, the total fluorescence intensity and the number of cells were quantified. The mean fluorescence intensity per cell was then derived (total intensity / cell count) to indicate the relative expression level of the target.

### Transmission electron microscopy (TEM)

To analyze macrophage mitochondria via TEM, cells subjected to different treatments were fixed in 2.5% glutaraldehyde buffer at 4 °C for 24 h, and washed with PBS. Samples were post-fixed in 1% osmium tetroxide for 1 h, dehydrated through a graded ethanol series, and embedded in epoxy resin. Ultrathin Sect.  (70 nm) were stained with uranyl acetate and lead citrate, then imaged under a Hitachi HT7700 TEM (Tokyo, Japan). Mitochondrial morphology was quantified in ImageJ using form factor analysis: perimeter² / (4π × area).

#### RNA-sequencing (RNA-seq)

BMDMs were stimulated with LPS and/or SKF for 12 h, followed by total RNA extraction using TRIzol reagent. RNA quality was confirmed by spectrophotometry and Agilent 2100 Bioanalyzer, with all samples showing good integrity (RNA integrity score > 7.0). Libraries of cDNA were constructed using the TruSeq Stranded mRNA LT Sample Prep Kit (Illumina, USA) and sequenced on the Illumina Novaseq 6000 platform (Macrogen, Korean) with 2 × 150 bp paired-end reads. Raw reads were filtered with fastp software, with quality checked simultaneously. Clean reads were aligned to the mouse GRCm39 genome via HISAT2, and transcripts were assembled with StringTie (default parameters). A merged transcriptome was generated using gffcompare. Gene expression was quantified as FPKM. Differentially expressed mRNAs (fold change > 2 or < 0.5; *p* < 0.05) were identified using edgeR, followed by GO and KEGG enrichment analyses.

### Seahorse metabolic analysis

Seahorse metabolic analysis was performed using an Agilent XFe96 analyzer (Agilent Technologies, USA) following the manufacturer’s protocol. In brief, BMDMs were seeded in XFe96 plates and stimulated under different conditions for 12 h. For mitochondrial respiration assessment, specific inhibitors and uncouplers were injected sequentially at defined time points: 2 µM oligomycin (to block ATP synthase), 1 µM carbonyl cyanide-4-trifluoromethoxy phenylhydrazone (FCCP, an uncoupler that maximizes oxygen consumption), and a 0.5 µM combination of rotenone and antimycin A (to fully inhibit the electron transport chain). Real-time oxygen consumption rate (OCR) was measured during these injections. For glycolytic function evaluation, extracellular acidification rate (ECAR) was determined. Prior to the assay, cells were washed and incubated for 1 h in XF assay medium (pH 7.4) lacking glucose, glutamine, and pyruvate. Cells were sequentially treated with 10 mM glucose (to initiate glycolysis), 1 µM oligomycin (to inhibit mitochondrial ATP production and emphasize glycolytic acidification), and 100 mM 2-deoxyglucose (2-DG, a glucose analog that inhibits glycolysis).

#### Statistics

Statistical analyses were performed using GraphPad Prism 8.0.1 (GraphPad Software, Inc., San Diego, CA), with the exception of RNA-seq data. Continuous variables underwent normality assessment via Shapiro-Wilk test. Group comparisons employed unpaired Student’s t-test (two-tailed), Mann-Whitney U-test, or one-way ANOVA with Bonferroni post-test. The categorical data between the DA-naïve and DA-treated patient groups were analyzed using the chi-square test. Results are presented as mean ± standard error of the mean (± SEM), with statistical significance defined as *p* < 0.05. All experiments were independently replicated at least three times.

## Supplementary Information

Supplementary Material 1.

## Data Availability

The datasets used and/or analyzed during the current study are available from the corresponding author on reasonable request.

## Abbreviations

ALI: Acute lung injury
ARDS: Acute respiratory distress syndrome
OXPHOS: Oxidative phosphorylation
ROS: Reactive oxygen species
CCL2: Chemokine (C-C motif) ligand 2
IL: Interleukin
TNF-α: Tumor necrosis factor-α
DA: Dopamine
β_2_AR: β_2_-adrenergic receptor
DRs: Dopamine receptors
D1R: D1 receptor
SKF: SKF38393
LPS: Lipopolysaccharide
RNA-seq: RNA sequencing
JAML: Junctional adhesion molecule-like protein
PBMCs: Peripheral blood mononuclear cells
DBH: DA β-hydroxylase
GEO: Gene Expression Omnibus
Th: Tyrosine hydroxylase
HVA: Homovanillic acid
VMA: Vanillylmandelic acid
DDC: DOPA decarboxylase
BALF: Bronchoalveolar lavage fluid
MPTP: 1-methyl-4-phenyl-1,2,3,6-tetrahydropyridine
Clo-lip: Clodronate liposome
BMDMs: Bone marrow-derived macrophages
Cab: Cabergoline
SCH: SCH23390
DCFH-DA: 2’,7’-dichlorodihydrofluorescein diacetate
TEM: Transmission electron microscopy
mROS: Mitochondrial reactive oxygen species
OCR: Oxygen consumption rate
KEGG: Kyoto Encyclopedia of Genes and Genomes
TLR: Toll-like receptor
GO: Gene ontology
WT: Wild type
rIL-10: Recombinant IL-10
Co-IP: Co-immunoprecipitation
cAMP: Cyclic adenosine monophosphate
JAMs: Junctional Adhesion Molecules
PBS: Phosphate-buffered saline
H&E staining: Hematoxylin and eosin staining
HVA: Homovanillic acid
VMA: Vanillylmandelic acid
UPLC-ESI-MS/MS: Ultra-performance liquid chromatography electrospray ionization-tandem mass spectrometry
qPCR: Quantitative-polymerase chain reaction
FCCP: Carbonyl cyanide-4-trifluoromethoxy phenylhydrazone
ECAR: Extracellular acidification rate
2-DG: 2-deoxyglucose
