# Supplementary material for: Dopamine signaling governs macrophage-mediated acute lung injury through JAML/IL-10-coupled mitochondrial regulation
Source: J Neuroinflammation. 2026 Apr 23;23:195. doi: 10.1186/s12974-026-03823-1 (PMC13248467; doi:10.1186/s12974-026-03823-1)
Supplement: Supplementary file 1 — Supplementary Material 1. [file 12974_2026_3823_MOESM1_ESM.docx]

**Supplementary Materials for**

**Dopamine signaling governs macrophage-mediated acute lung injury through JAML/IL-10-coupled mitochondrial regulation**

**
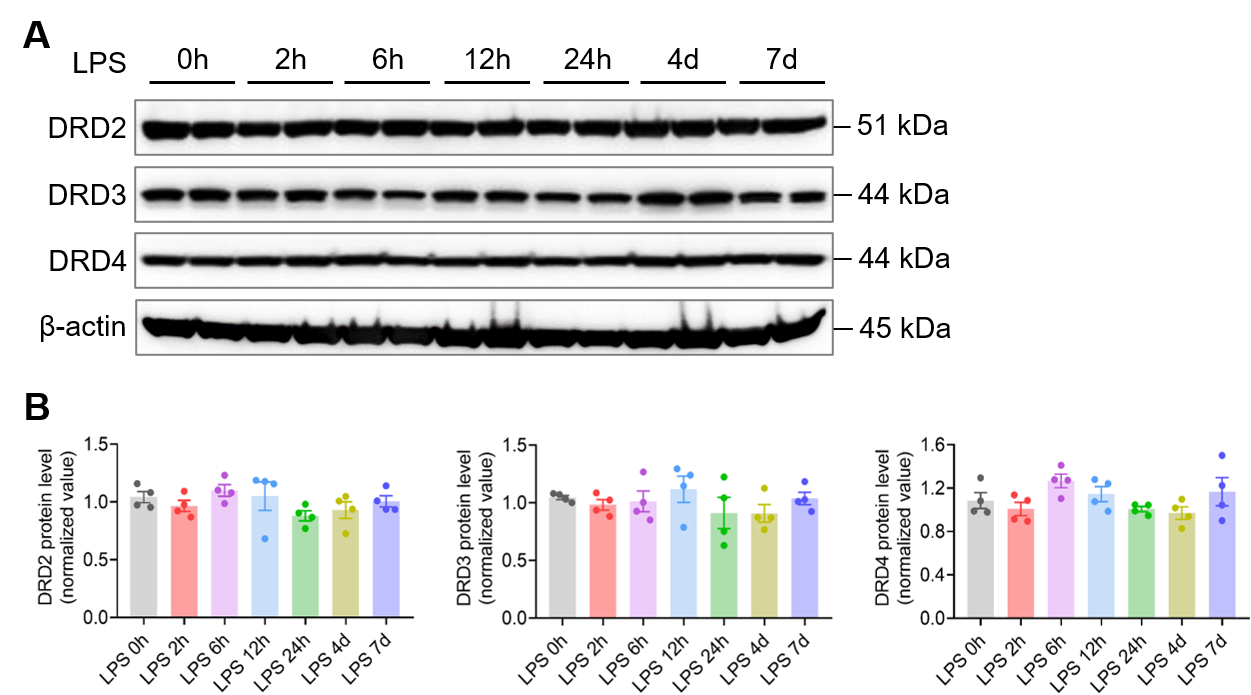
**

**Supplementary Figure 1. (A, B)** Immunoblot images **(A)** and the quantitative analysis **(B)** of DRD2, DRD3 and DRD4 expression in the lung tissues of indicated groups. n = 4 in each group. All samples were biologically independent and three or more independent experiments with similar results were performed (“n” represents the number of independent biological replicates). Data are presented as mean ± SEM and analyzed with a 95% confidence interval. Statistical analysis was performed via one-way ANOVA followed by Bonferroni’s post hoc test.


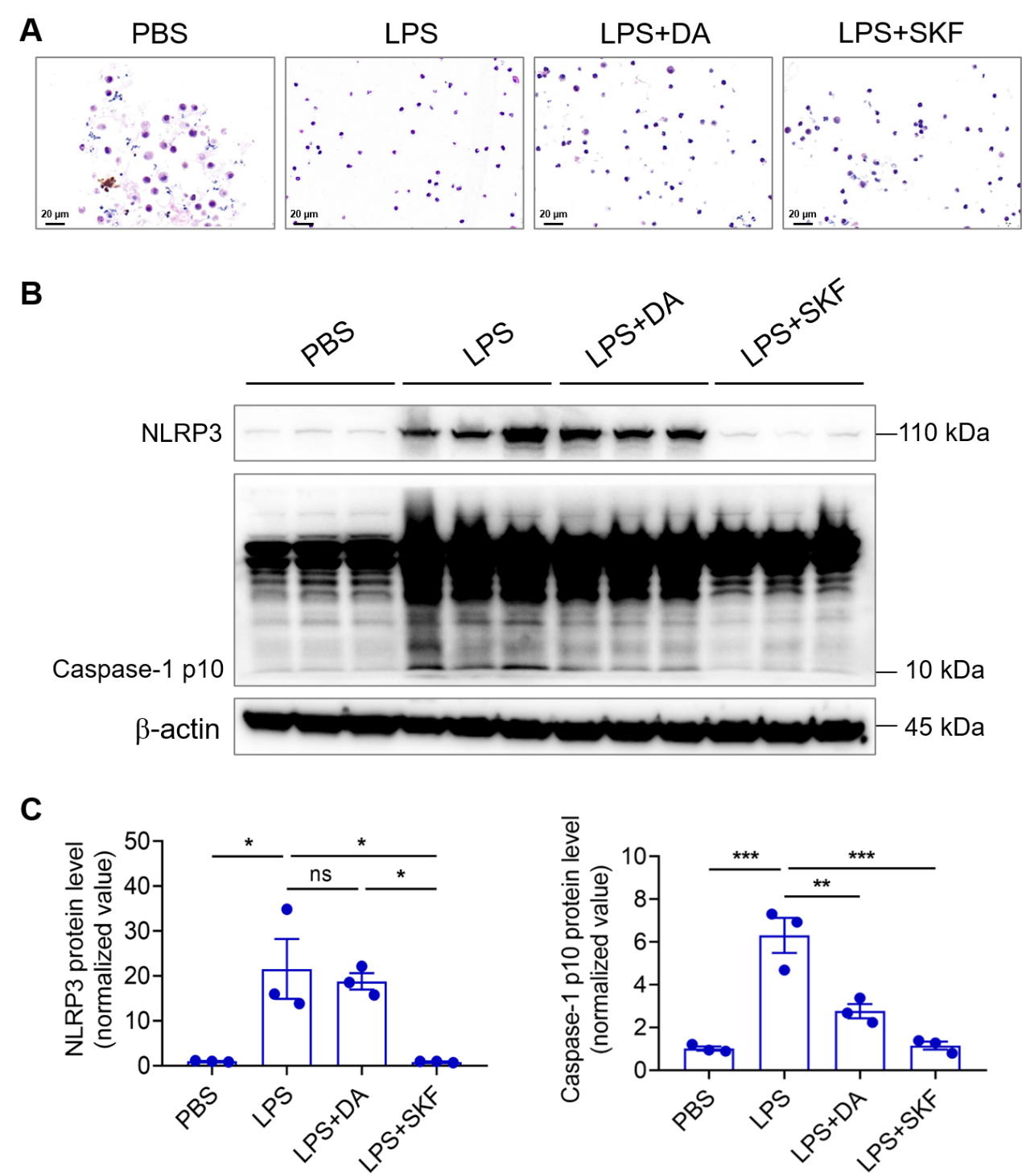


**Supplementary Figure 2.** **(A)** Representative images of Wright-Giemsa staining in BALF samples from mice of indicated group. Scale bar: 20 µm. **(B, C)** Immunoblot images **(B)** and the quantitative analysis **(C)** of NLRP3 and Caspase-1 p10 expression in the lung tissues of indicated groups. n = 3 in each group. All samples were biologically independent and three or more independent experiments with similar results were performed (“n” represents the number of independent biological replicates). Data are presented as mean ± SEM and analyzed with a 95% confidence interval. Statistical analysis was performed via one-way ANOVA followed by Bonferroni’s post hoc test. *p < 0.05, **p < 0.01, ***p < 0.001. ns, not significant.


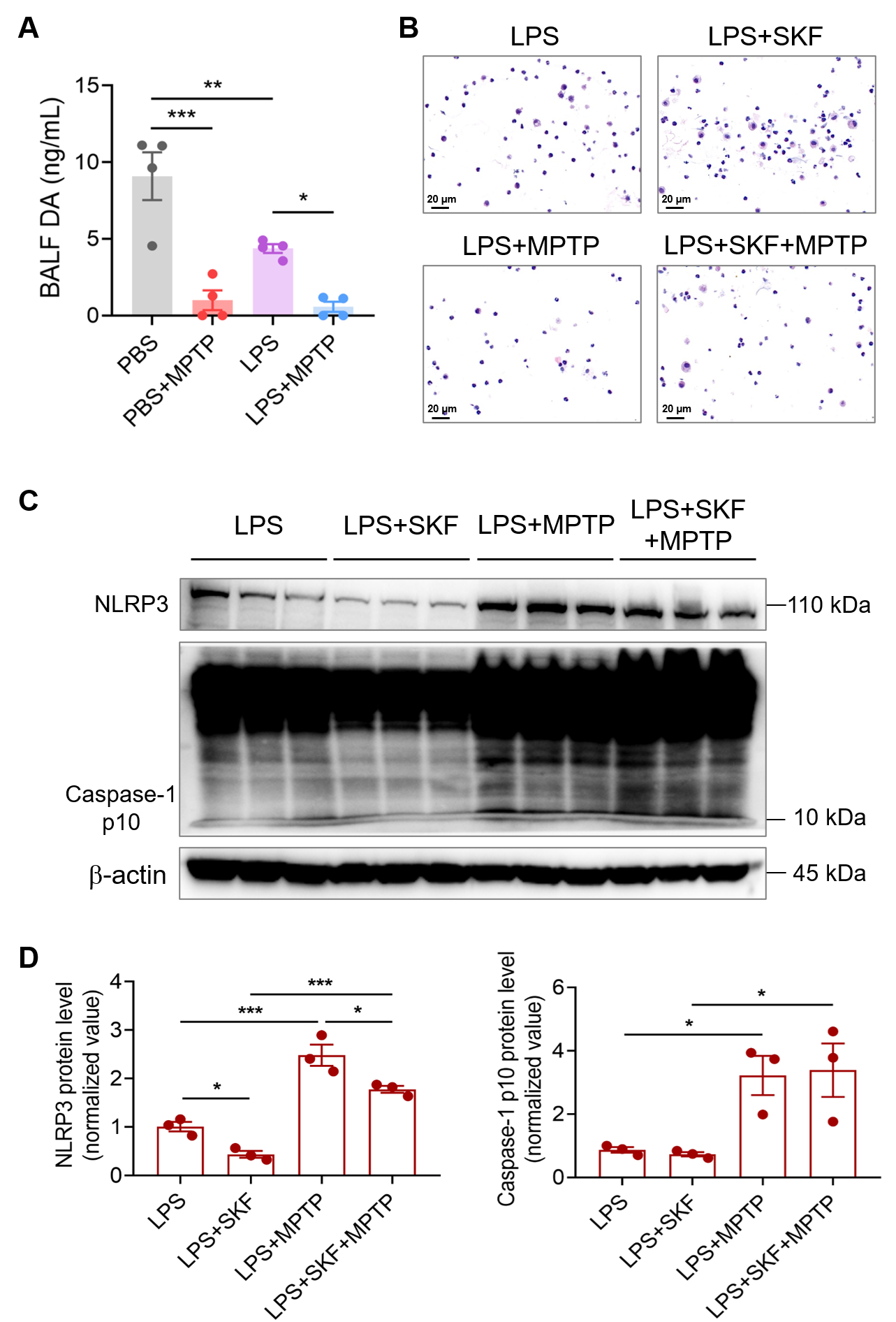


**Supplementary Figure 3. (A)** DA level in the BALF of mouse from different groups (n = 4 in each group). **(B)** Representative images of Wright-Giemsa staining in BALF samples from mice of indicated group. Scale bar: 20 µm. **(C, D)** Immunoblot images **(C)** and the quantitative analysis **(D)** of NLRP3 and Caspase-1 p10 expression in the lung tissues of indicated groups. n = 3 in each group. All samples were biologically independent and three or more independent experiments with similar results were performed (“n” represents the number of independent biological replicates). Data are presented as mean ± SEM and analyzed with a 95% confidence interval. Statistical analysis was performed via one-way ANOVA followed by Bonferroni’s post hoc test. *p < 0.05, ***p < 0.001.

**
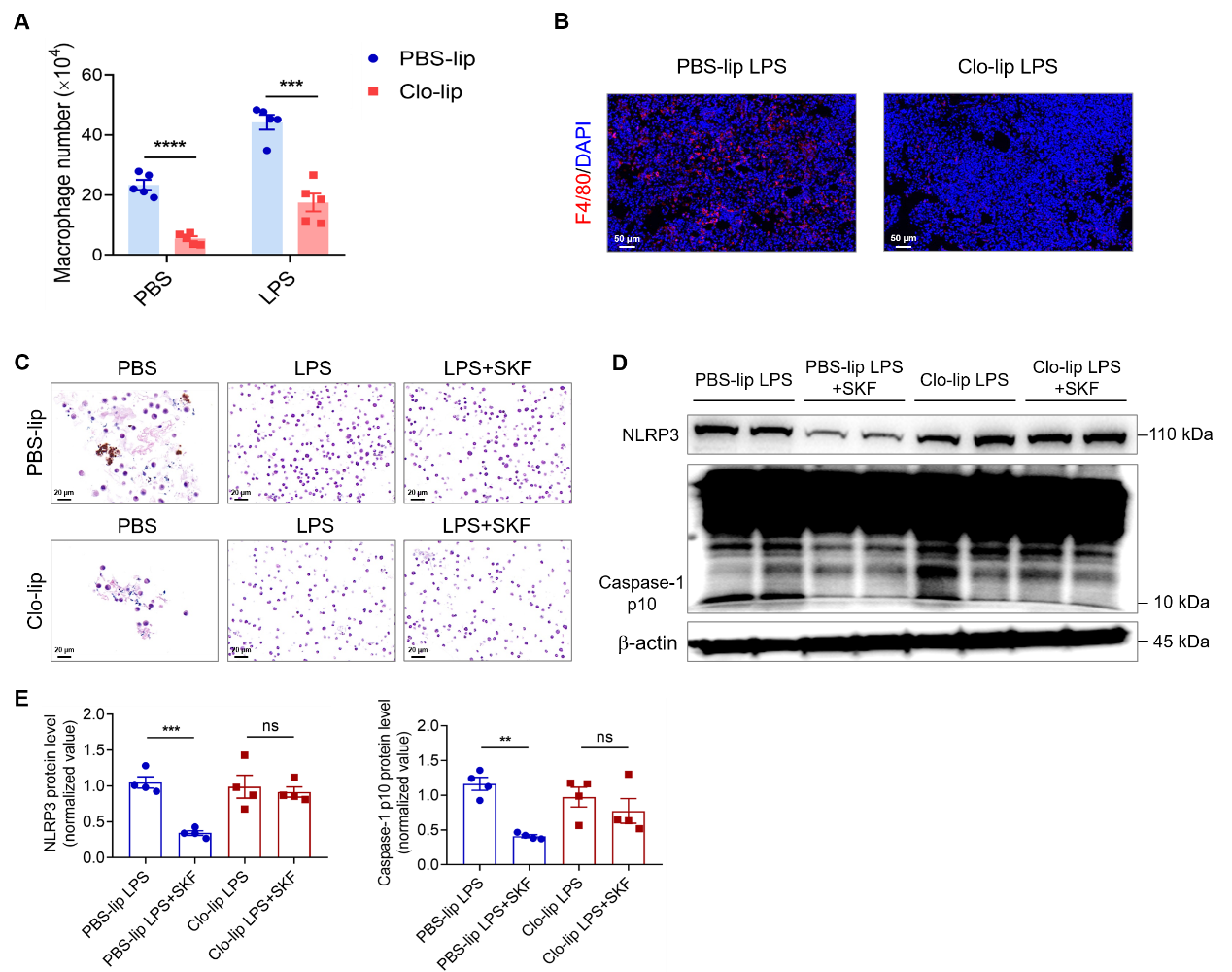
**

**Supplementary Figure 4. (A)** BALF macrophage count in mice with distinct treatments (n = 5 in each group). **(B)** Representative immunofluorescence images of macrophages in mouse lung stained with F4/80. Nuclei were stained with DAPI, displayed in blue. Scale bar: 50 µm. **(C)** Representative images of Wright-Giemsa staining in BALF samples from mice of indicated group. Scale bar: 20 µm. **(D, E)** Immunoblot images **(D)** and the quantitative analysis **(E)** of NLRP3 and Caspase-1 p10 expression in the lung tissues of distinct groups. n = 4 in each group. All samples were biologically independent and three or more independent experiments with similar results were performed (“n” represents the number of independent biological replicates). Data are presented as mean ± SEM and analyzed with a 95% confidence interval. Statistical analysis was performed using two-tailed unpaired Student t test or one-way ANOVA followed by Bonferroni’s post hoc test. **p < 0.01, ***p < 0.001, ****p < 0.0001, ns, not significant.


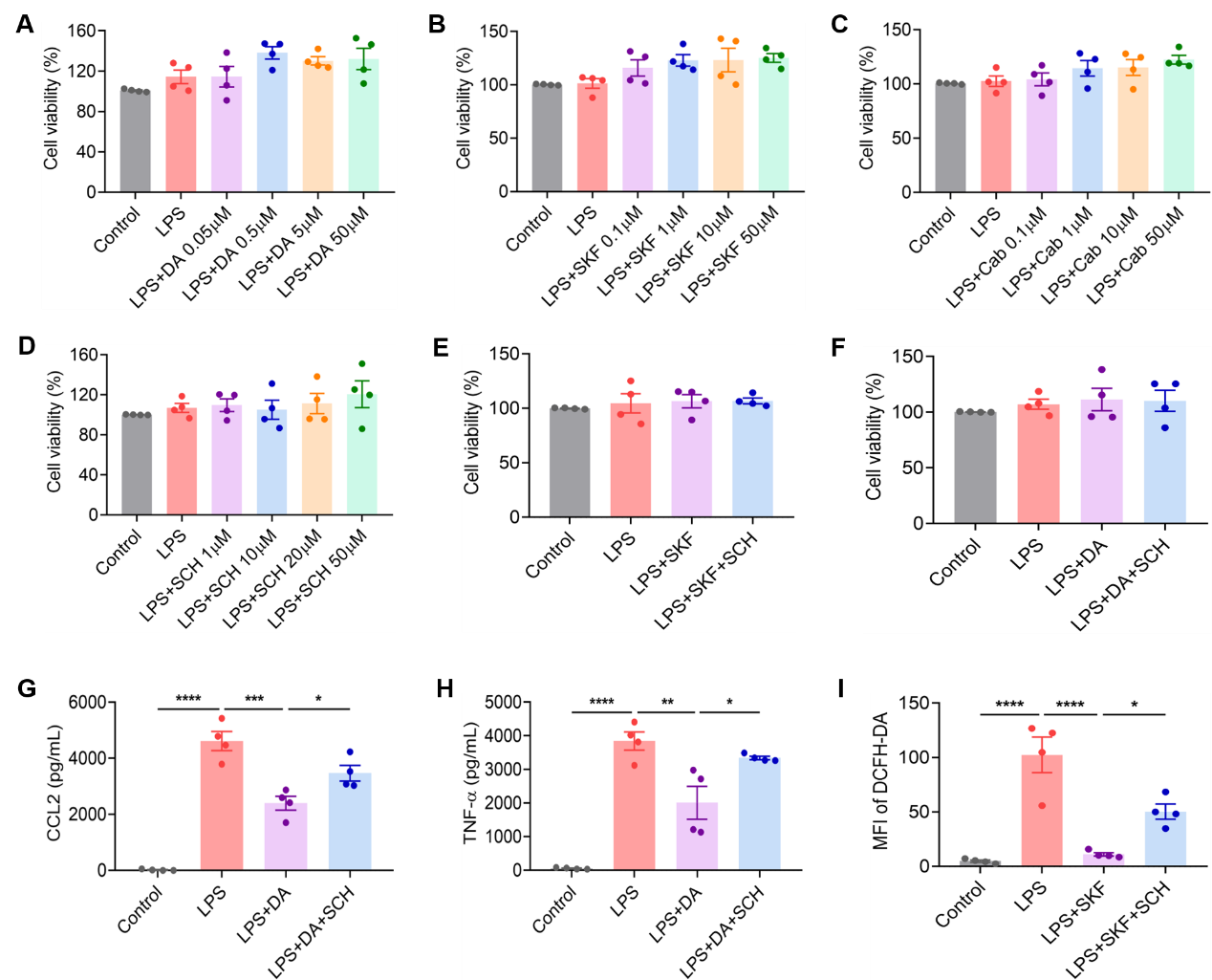


**Supplementary Figure 5. (A-F)** Cell viability of BMDMs was measured under treatments of different concentrations of DA (**A**, n = 4 in each group), SKF (**B**, n = 4 in each group), Cab (**C**, n = 4 in each group), SCH (**D**, n = 4 in each group), SKF combined with SCH (**E,** n = 4 in each group), or DA combined with SCH (**F,** n = 4 in each group). **(G, H)** CCL2 **(G)** and TNF-α **(H)** were detected in the supernatants of BMDMs from indicated groups (n = 4 in each group). **(I)** Analysis of mean fluorescence intensity (MFI) of DCFH-DA in BMDMs from different treatment groups. All samples were biologically independent and three or more independent experiments with similar results were performed (“n” represents the number of independent biological replicates). Data are presented as mean ± SEM and analyzed with a 95% confidence interval. Statistical analysis was performed using one-way ANOVA followed by Bonferroni’s post hoc test. *p < 0.05, **p < 0.01, ***p < 0.001, ****p < 0.0001.


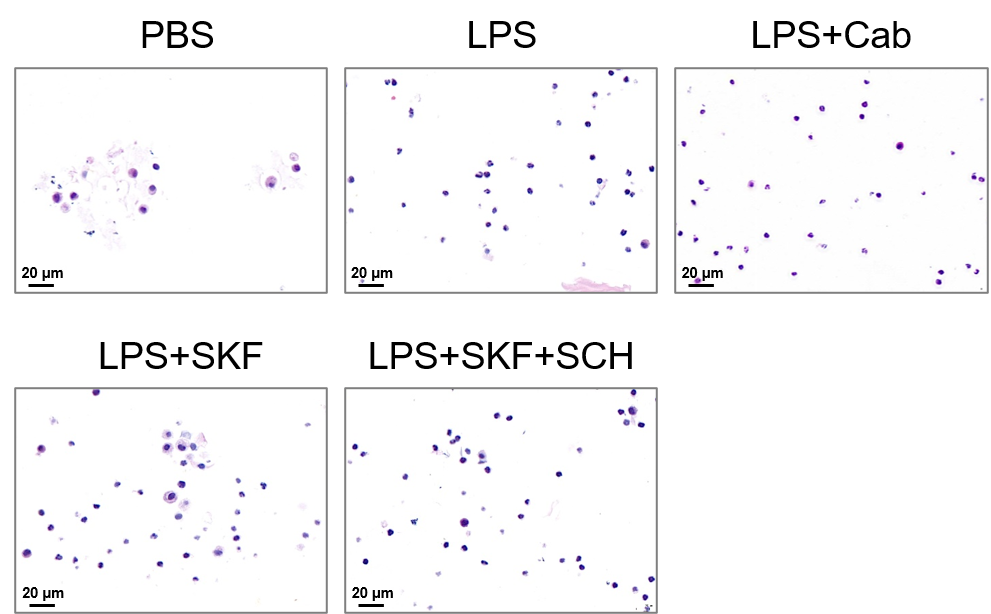


**Supplementary Figure 6.** Representative images of Wright-Giemsa staining in BALF samples from mice of indicated group. Scale bar: 20 µm.


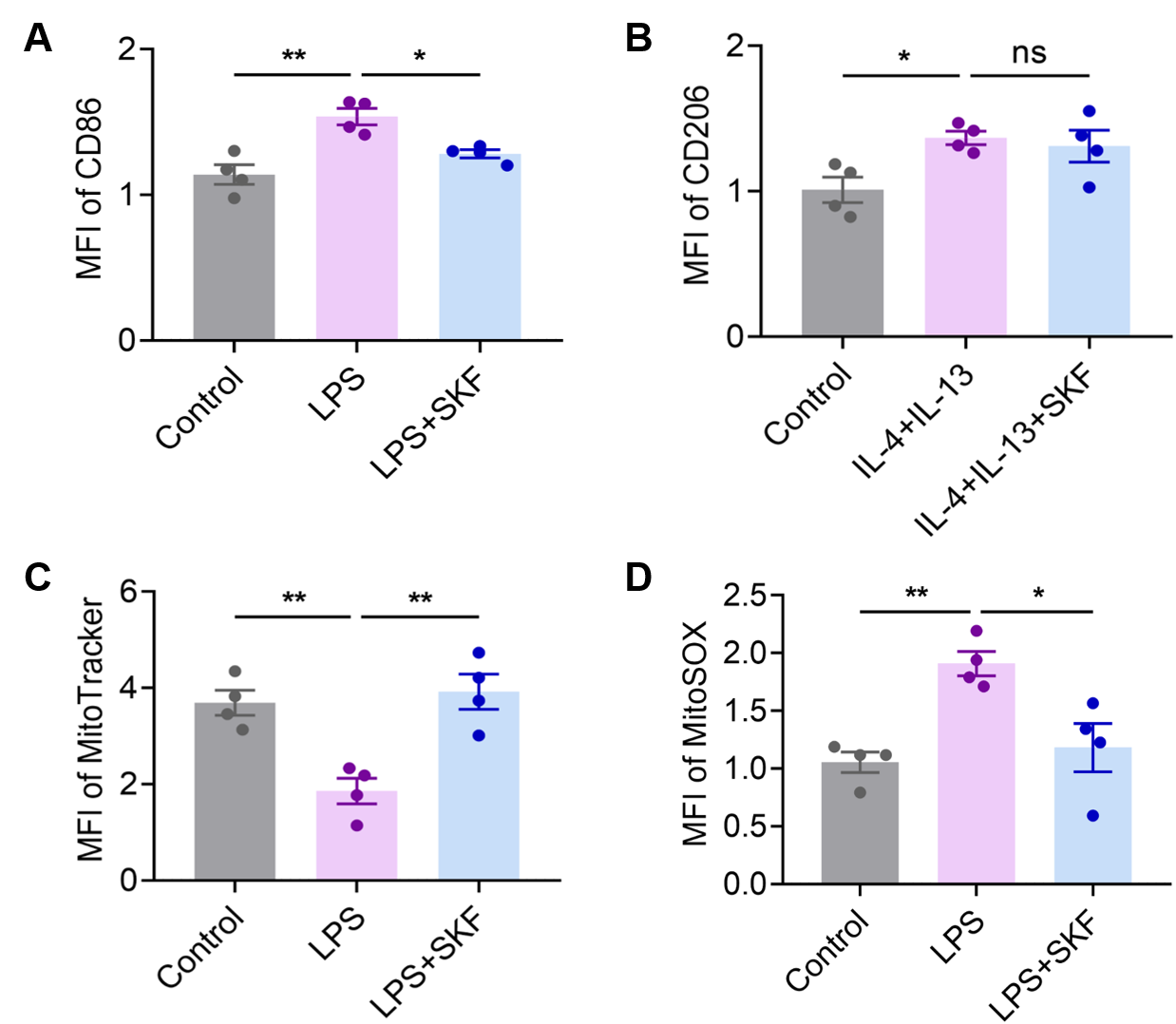


**Supplementary Figure 7. (A, B)** Analysis of MFI of CD68 **(A**, n = 4 in each group**)** and CD206 **(B**, n = 4 in each group**)** in BMDMs from different treatment groups. **(C, D)** Analysis of MFI of MitoTracker **(C**, n = 4 in each group**)** and MitoSOX **(D**, n = 4 in each group**)** in BMDMs from different treatment groups. All samples were biologically independent and three or more independent experiments with similar results were performed (“n” represents the number of independent biological replicates). Data are presented as mean ± SEM and analyzed with a 95% confidence interval. Statistical analysis was performed using one-way ANOVA followed by Bonferroni’s post hoc test. *p < 0.05, **p < 0.01, ns, not significant.

**
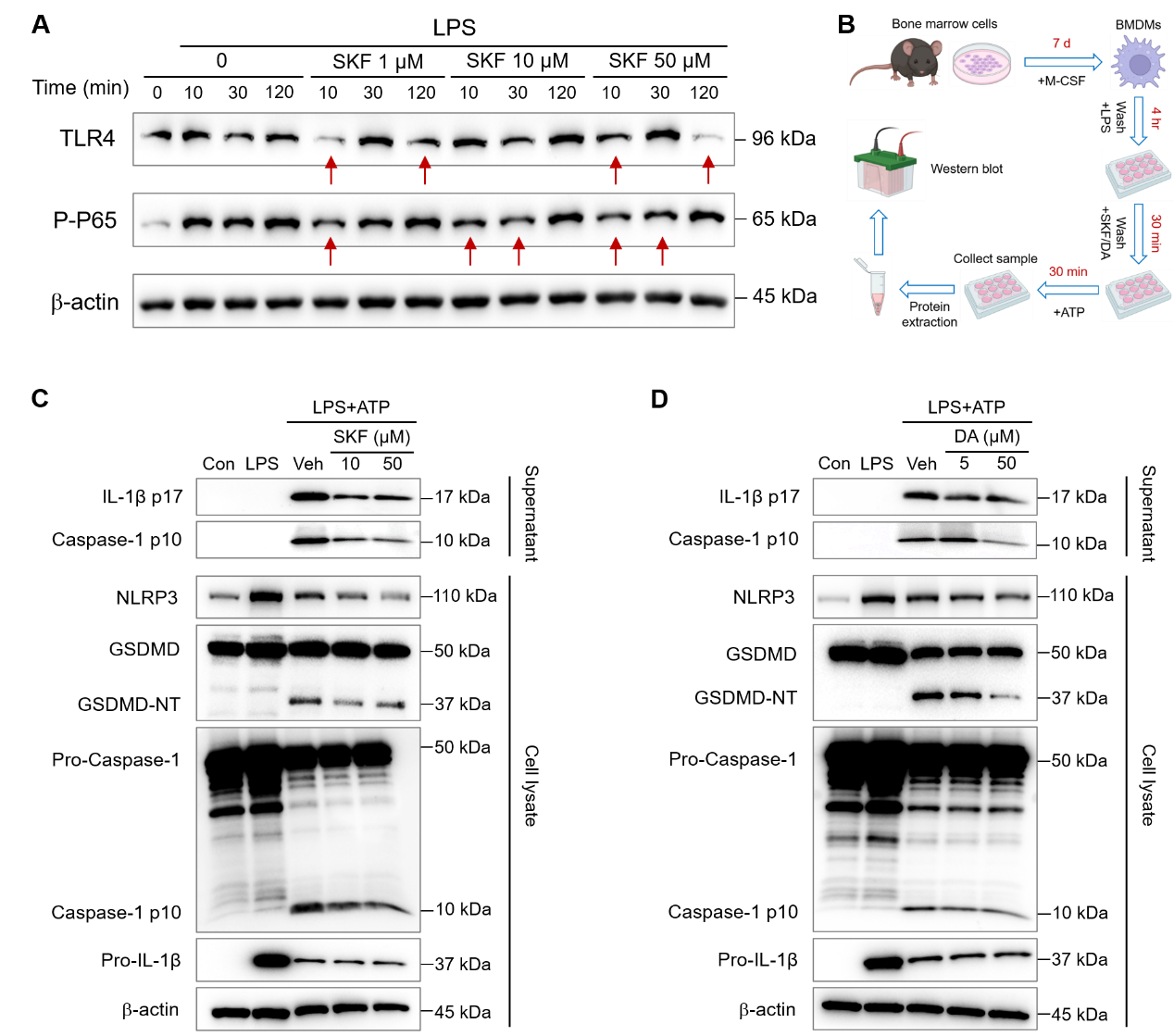
**

**Supplementary Figure 8. (A)** Representative immunoblot images of TLR4 and p-P65 expression in BMDMs under distinct treatments at different time points. Red arrows indicate protein expression inhibited by SKF. **(B)** Schematic diagram of NLRP3 inflammasome activation in BMDMs primed with LPS and activated with ATP. Proteins were extracted from both cells and supernatants for immunoblot analysis. **(C, D)** Immunoblot analysis of multiple key endpoints of inflammasome activation, including Caspase-1 activation, Gasdermin-D (GSDMD) cleavage and IL-1β maturation and secretion in the presence of SKF **(C)** or DA **(D)** at indicated concentrations.

**
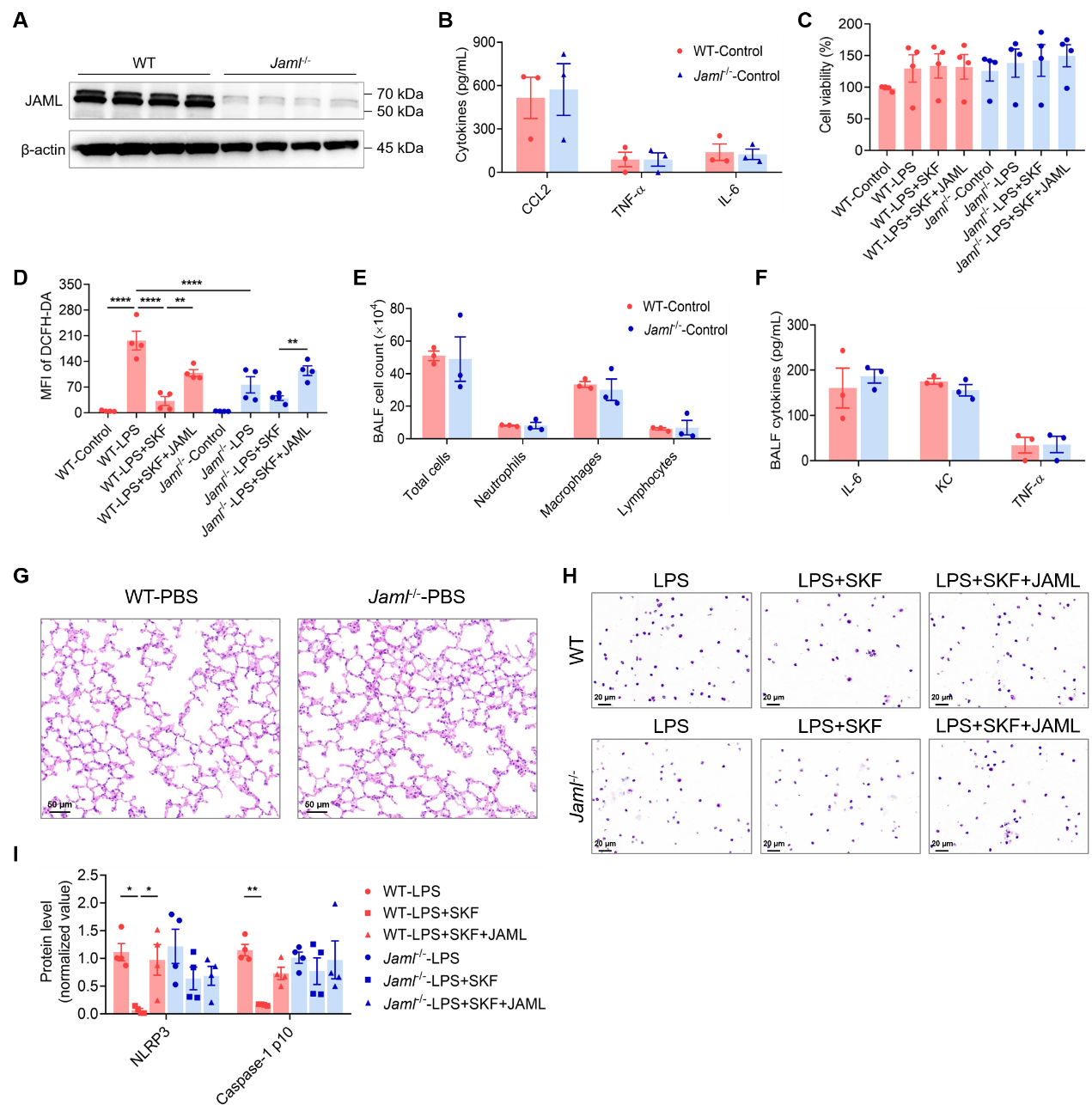
**

**Supplementary Figure 9. (A)** Immunoblot image of JAML in BMDMs from wild-type (WT) and *Jaml* knockout (*Jaml^-/-^*) mice (n = 4 in each group). **(B)** Cytokines release of BMDMs from WT and *Jaml^-/-^* mice under unstimulated condition (n = 3 in each group). **(C)** Cell viability of BMDMs was measured under different treatments (n = 4 in each group). **(D)** Analysis of MFI of DCFH-DA in BMDMs from different treatment groups (n = 4 in each group). **(E, F)** Airway inflammatory cell counts **(E)** and pro-inflammatory cytokine levels **(F)** in WT and *Jaml^-/-^* mice under physiological condition (n = 3 in each group). **(G)** Representative H&E staining images of lung tissues from WT and *Jaml^-/-^* mice under physiological condition. Scale bar: 50 µm. **(H)** Representative images of Wright-Giemsa staining in BALF samples from mice of indicated group. Scale bar: 20 µm. **(I)** The quantitative analysis of immunoblotted NLRP3 and Caspase-1 p10 proteins in the mouse lung of distinct groups (n = 4 in each group). All samples were biologically independent and three or more independent experiments with similar results were performed (“n” represents the number of independent biological replicates). Data are presented as mean ± SEM and analyzed with a 95% confidence interval. Statistical analysis was performed using one-way ANOVA followed by Bonferroni’s post hoc test. *p < 0.05, **p < 0.01, ****p < 0.0001.

**
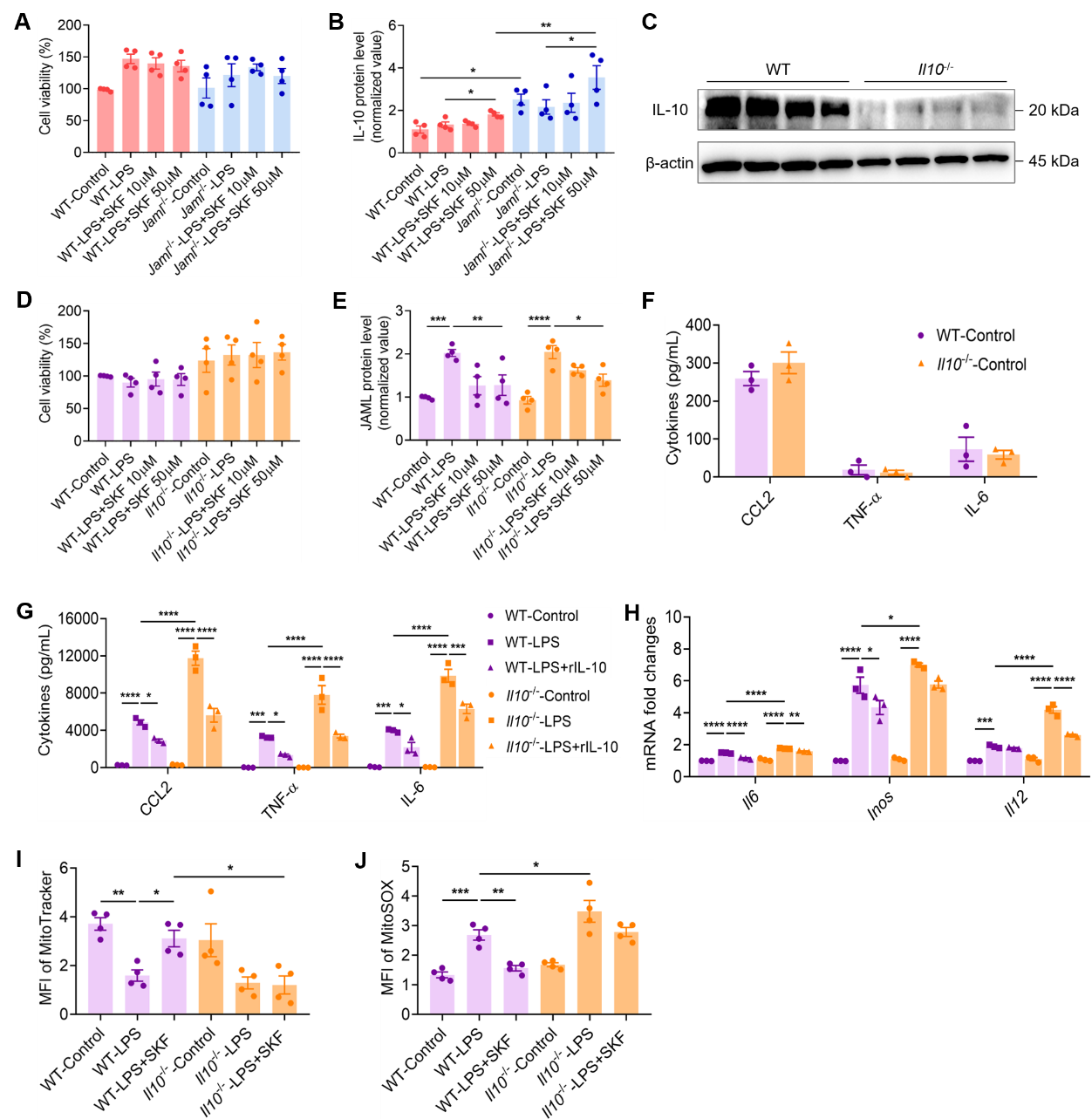
**

**Supplementary Figure 10. (A)** Cell viability of BMDMs was measured under different treatments (n = 4 in each group). **(B)** The quantitative analysis of immunoblotted IL-10 protein in BMDMs with distinct interventions (n = 4 in each group). **(C)** Immunoblot image of IL-10 in BMDMs from WT and *Il-10^-/-^* mice (n = 4 in each group). **(D)** Cell viability of BMDMs was measured under different treatments (n = 4 in each group). **(E)** The quantitative analysis of immunoblotted JAML protein in BMDMs with distinct interventions (n = 4 in each group). **(F)** Cytokines release of BMDMs from WT and *Il10^-/-^* mice under unstimulated condition (n = 3 in each group). **(G, H)** Effects of recombinant exogenous IL-10 (rIL-10) on pro-inflammatory cytokine secretion **(G)** and M1 marker expression **(H)** in BMDMs derived from WT and *Il-10^-/-^* mice. **(I, J)** Analysis of MFI of MitoTracker **(I**, n = 4 in each group**)** and MitoSOX **(J**, n = 4 in each group**)** in BMDMs from different treatment groups. All samples were biologically independent and three or more independent experiments with similar results were performed (“n” represents the number of independent biological replicates). Data are presented as mean ± SEM and analyzed with a 95% confidence interval. Statistical analysis was performed using one-way ANOVA followed by Bonferroni’s post hoc test. *p < 0.05, **p < 0.01, ***p < 0.001, ****p < 0.0001.

**
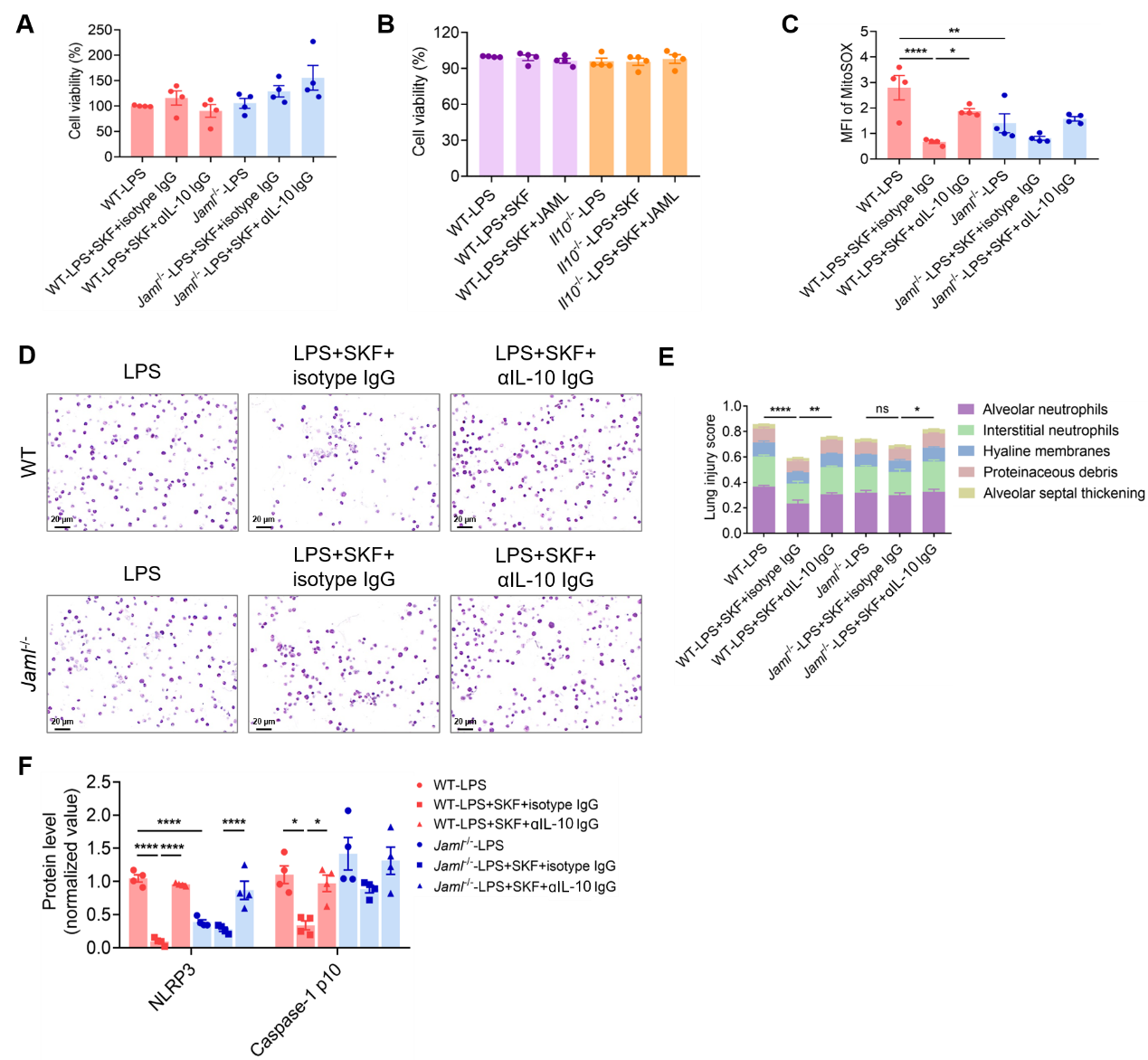
Supplementary Figure 11. (A, B)** Cell viability of BMDMs was measured under different treatments (n = 4 in each group). **(C)** Analysis of MFI of MitoSOX **(**n = 4 in each group**)** in BMDMs from different treatment groups. **(D)** Representative images of Wright-Giemsa staining in BALF samples from mice of indicated group. Scale bar: 20 µm. **(E)** The injury score analysis of H&E-stained lung tissues was performed at 24 hours after LPS (n = 5 in each group). **(F)** The quantitative analysis of immunoblotted NLRP3 and Caspase-1 p10 proteins in lung tissues of mice with distinct interventions (n = 4 in each group). All samples were biologically independent and three or more independent experiments with similar results were performed (“n” represents the number of independent biological replicates). Data are presented as mean ± SEM and analyzed with a 95% confidence interval. Statistical analysis was performed using one-way ANOVA followed by Bonferroni’s post hoc test. *p < 0.05, **p < 0.01, ****p < 0.0001, ns, not significant.

**
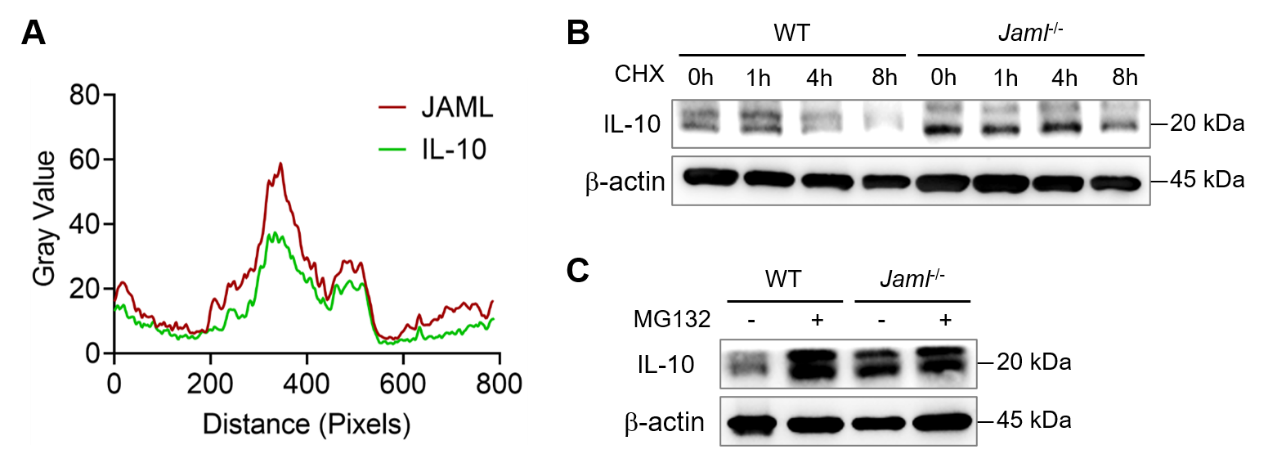
**

**Supplementary Figure 12. (A)** Co-localization analysis of JAML and IL-10 immunofluorescence staining of mouse lung by ImageJ. **(B)** Immunoblot analysis of IL-10 in BMDMs from WT and *Jaml*^⁻/⁻^ mice treated with cycloheximide (CHX, 20 μg/mL) and collected at the indicated time points (0, 1, 4, and 8 h) (n = 3 in each group). **(C)** Immunoblot analysis of IL-10 in BMDMs from WT and *Jaml*^⁻/⁻^ mice treated with MG132 (10 μM) and collected at 0 and 6 h (n = 3 in each group). All samples were biologically independent and three or more independent experiments with similar results were performed (“n” represents the number of independent biological replicates).

**
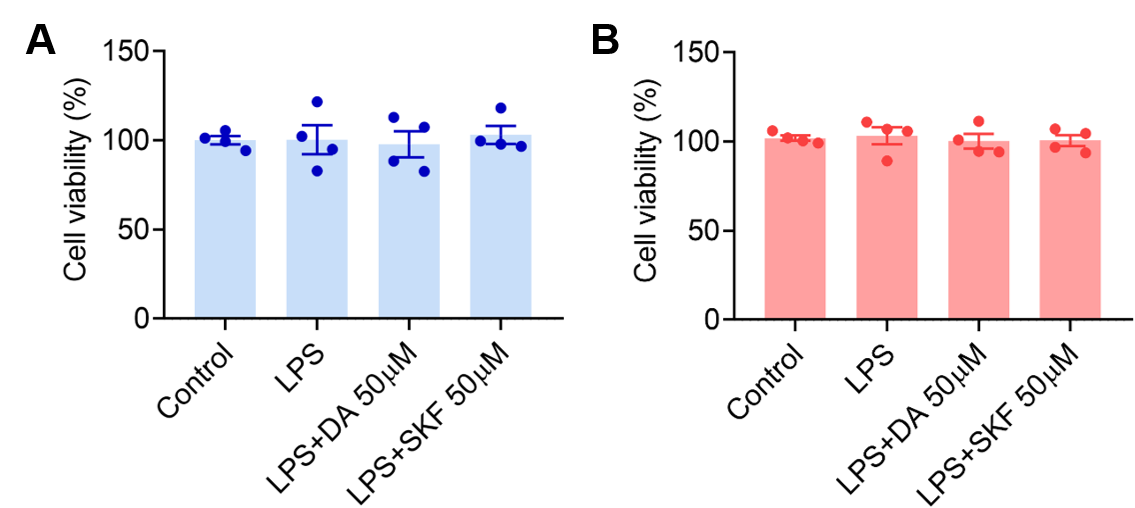
**

**Supplementary Figure 13. (A, B)** Cell viability of BMDMs was measured under different treatments (both n = 4 in each group). All samples were biologically independent and three or more independent experiments with similar results were performed (“n” represents the number of independent biological replicates). Data are presented as mean ± SEM and analyzed with a 95% confidence interval. Statistical analysis was performed using one-way ANOVA followed by Bonferroni’s post hoc test.


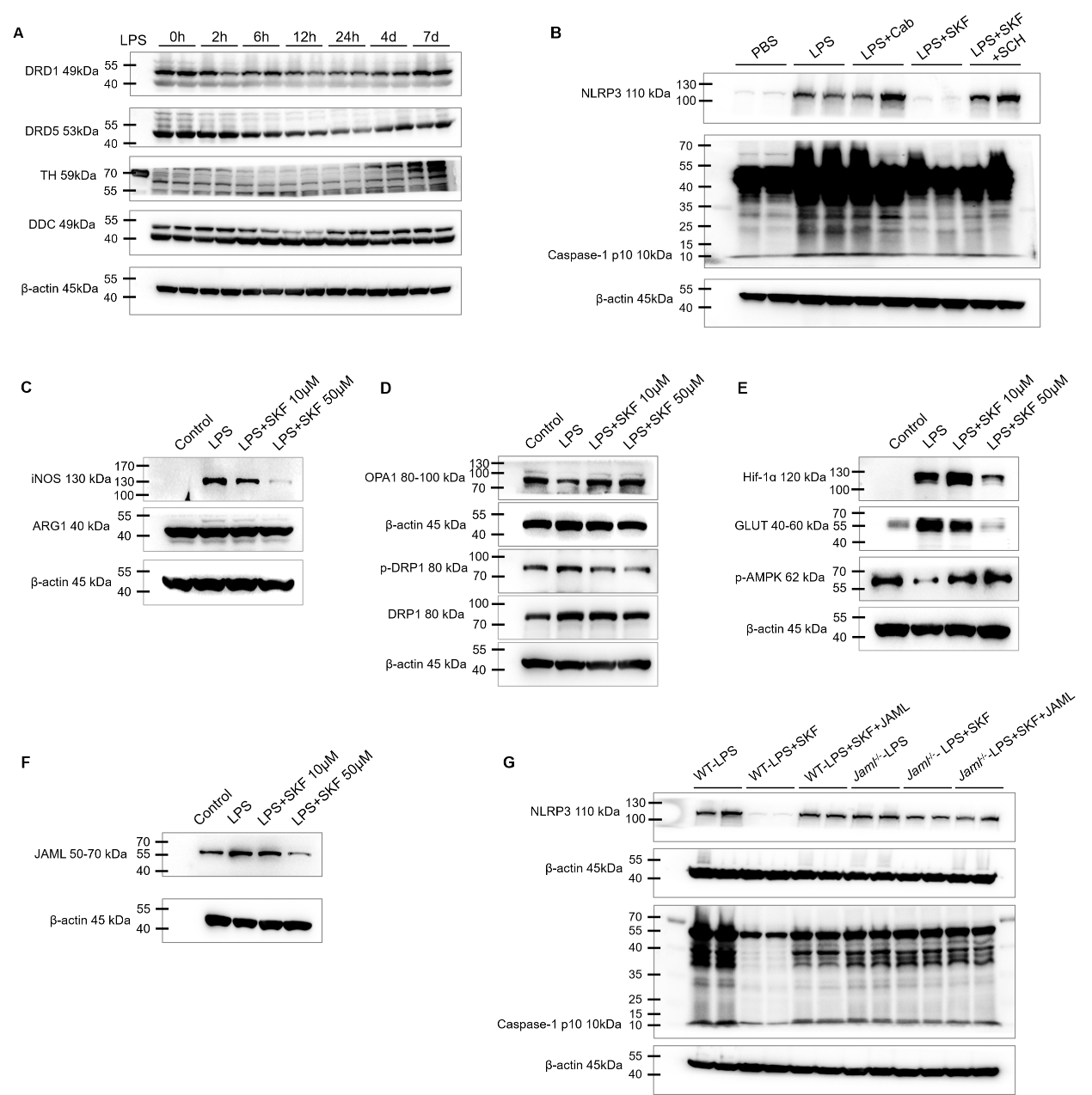


**Supplementary Figure 14. (A-G)** The uncropped immunoblot images corresponding to Figure 1H **(A)**, Figure 3N **(B)**, Figure 4C, I, N **(C-E)**, and Figure 5E, N **(F, G)** in the original text.


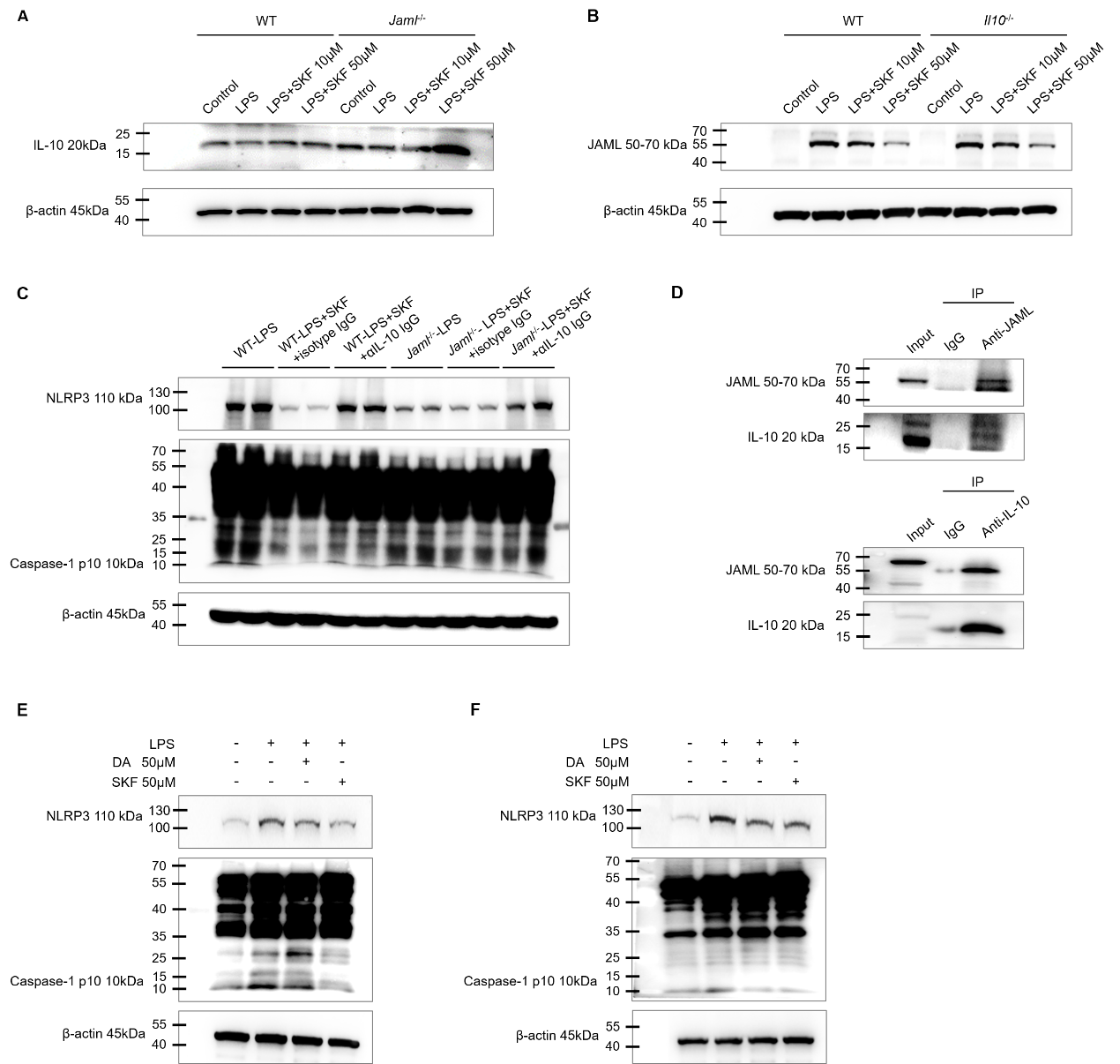


**Supplementary Figure 15. (A-F)** The uncropped immunoblot images corresponding to Figure 6B, D **(A, B)**, Figure 7I, K **(C, D)**, and Figure 8C, E **(E, F)** in the original text.

**Table S1.** Demographic and clinical characteristics of 6 ARDS patients and 4 healthy volunteers.

| **Group** | **ARDS** | | | | | | **Healthy Control** | | | |
| --- | --- | --- | --- | --- | --- | --- | --- | --- | --- | --- |
| **Case** | **Case 1** | **Case 2** | **Case 3** | **Case 4** | **Case 5** | **Case 6** | **Case 7** | **Case 8** | **Case 9** | **Case 10** |
| **Age (years)** | 69 | 81 | 92 | 83 | 76 | 58 | 66 | 37 | 38 | 57 |
| **Sex** | F | F | M | F | M | M | M | M | F | F |
| **Smoking** | **√** | **×** | **×** | **×** | **×** | **√** | **×** | **√** | **×** | **×** |
| **Pack-years, Yrs** | 60 | - | - | - |  | 40 | - | 10 | - | - |
| **Cancer history** | **√** | **×** | **√** | **×** | **×** | **×** | **×** | **×** | **×** | **×** |
| **Sample** | Peripheral blood | | | | | | | | | |
| **Underlying diseases** | | | | | | | | | | |
| Hypertension | **×** | **√** | **√** | **√** | **√** | **√** | **×** | **×** | **×** | **×** |
| Diabetes | **×** | **√** | **√** | **×** | **√** | **×** | **×** | **×** | **×** | **×** |
| Cardiac disease | **√** | **×** | **√** | **√** | **√** | **√** | **×** | **×** | **×** | **×** |
| Autoimmune diseases | **×** | **×** | **√** | **×** | **×** | **×** | **×** | **×** | **×** | **×** |
| Others | **×** | **×** | **×** | **×** | **×** | **√** | **×** | **×** | **×** | **×** |
| **Outcome** | | | | | | | | | | |
| Survival | **√** | **×** | **√** | **√** | **√** | **√** | **√** | **√** | **√** | **√** |

**Table S2.** Clinical characteristics of ARDS patients with or without clinical usage of DA hydrochloride.

|  | **DA-naïve patients** (n=53) | **DA-treated patients** (n=28) | **χ²** | ***p* value** |
| --- | --- | --- | --- | --- |
| **Age**（$\bar{x}$*± s*, year） | 69.62 ± 13.65 | 66.32 ± 18.08 | - | *0.468* |
| **Sex** |  |  |  |  |
| Female | 22 | 9 | *0.029* | *0.869* |
| Male | 31 | 19 | - | *-* |
| PaO_2_ | 82.64 ± 47.95 | 88.18 ± 27.98 | - | *0.334* |
| PaCO_2_ | 35.85 ± 9.78 | 43.18 ± 13.84 | - | *0.120* |
| **Cause of ARDS** |  |  |  |  |
| Pneumonia | 25 | 11 | *0.475* | *0.491* |
| Sepsis | 9 | 2 | *1.518* | *0.218* |
| Abdominal diseases | 3 | 3 | *0.683* | *0.409* |
| Trauma | 3 | 1 | *0.170* | *0.680* |
| Ohters | 13 | 11 | *1.959* | *0.162* |
| **Hypertension** | 20 | 12 | *0.211* | *0.647* |
| **Diatebes** | 8 | 4 | *0.008* | *0.928* |
| **Heart diseases** | 13 | 8 | *0.122* | *0.726* |
| **Death** | 15 | 13 | *2.457* | *0.117* |

Data are presented as mean ± SEM and analyzed with a 95% confidence interval. Statistical analysis was performed using χ² test.
